# Taxonomic characterization of *Sphaerotilus microaerophilus* sp. nov., a sheath-forming microaerophilic bacterium of activated sludge origin

**DOI:** 10.1007/s00203-024-03991-9

**Published:** 2024-05-10

**Authors:** Shiori Narihara, Shun Chida, Naoki Matsunaga, Ryosuke Akimoto, Mizuki Akimoto, Aoi Hagio, Tomomi Mori, Tadashi Nittami, Michio Sato, Sehui Mun, Hyeonjin Kang, Ji Hwan Back, Minoru Takeda

**Affiliations:** 1 Graduate School of Engineering, Yokohama National University, Tokiwadai 79-5, Hodogaya, Yokohama, 240-8501 Japan; 2 School of Agriculture, Meiji University, 1-1-1 Higashimita, Tama, Kawasaki, 214-8571 Japan; 3 Department of Food Science and Nutrition, Gwangju University, 277, Hyodeok-Ro, Nam-Gu, Gwangju, 61743 Korea

**Keywords:** *Sphaerotilus microaerophilus* sp. nov., Sheath, Microaerophile, Activated sludge

## Abstract

A microaerophilic Gram-stain-negative bacilliform bacterial strain, FB-5^ T^, was isolated from activated sludge in Yokohama, Japan, that exhibited filamentous growth and formed a microtube (sheath). Cells were motile using a single polar flagellum. The optimum growth temperature and pH were 30 °C and 7.5, respectively. Strain FB-5^ T^ was catalase-negative. Peptides and amino acids were utilized as energy and carbon sources. Sugars and organic acids were not utilized. Vitamin B_12_ enhanced the growth of strain FB-5^ T^. Sulfur-dependent lithotrophic growth was possible. Major respiratory quinone was UQ-8. Major fatty acids were C_16:1_ω7 and C_16:0_. The genomic DNA G + C content was 69.16%. Phylogenetic analysis of the 16S rRNA gene suggested that strain FB-5^ T^ belongs to the genus *Sphaerotilus*. The close relatives were *S. natans* subsup. *sulfidivorans* and *S. natans* subsup. *natans* with 98.0% and 97.8% similarity based on the 16S rRNA gene analysis, respectively. The genome size (6.06 Mbp) was larger than that (4.39–5.07 Mbp) of the *Sphaerotilus* strains. The AAI values against the related strains ranged from 71.0 to 72.5%. The range of ANI values was 81.7 − 82.5%. In addition to these distinguishable features of the genome, the core genome and dDDH analyses suggested that this strain is a novel member of the genus *Sphaerotilus*. Based on its physiological properties and genomic features, strain FB-5^ T^ is considered as a novel species of the genus *Sphaerotilus*, for which the name *S. microaerophilus* sp. nov. is proposed. The type strain is FB-5^ T^ (= JCM 35424^ T^ = KACC 23146^ T^).

**Supplementary Information:**

The online version contains supplementary material available at 10.1007/s00203-024-03991-9.

## Introduction

Bacterial strains of the genera *Sphaerotilus* and *Leptothrix* are sheath-forming bacteria within the class *Betaproteobacteria*, which are collectively called the *Sphaerotilus-Leptothrix* group due to their morphological, physiological, and phylogenetic relationships (van Veen et al. 1978; Siering and Ghiorse 1996). The *Sphaerotilus-Leptothrix* group belongs to the family *Comamonadaceae* of the order *Burkholderiales* (Willems et al. 1991)*.* Recently, the family *Sphaerotilaceae,* which is closely related to the family *Comamonadaceae* and consists of members of the *Sphaerotilus-Leptothrix* group and related genera, has been approved by the International Committee on Systematics of Prokaryotes (ICSP) (Liu et al. 2022; Oren and Göker 2023). Members of the *Sphaerotilus-Leptothrix* group are widely distributed in aquatic environments including streams, springs, and activated sludge (van Veen et al. 1978; Emerson and Ghiorse 1992; Gaval et al. 2003; Baskar et al. 2012; Schmidt et al. 2014). The genus *Sphaerotilus* comprises four species, *Sphaerotilus mobilis* (formerly *Leptothrix mobilis*)*, Sphaerotilus hippei*, *Sphaerotilus montanus*, and *Sphaerotilus natans* (further classified as *S. natans* subsp. *natans* and *S. natans* subsp. *sulfidivorans*) for which type strains are available (Gridneva et al. 2011; Liu et al. 2022). Reclassification of *S. natans* subsp. *sulfidivorans* as *Sphaerotilus sulfidivorans* has been proposed in 2021 (Grabovich et al. 2021). Currently, *S. sulfidivorans* is a synonym of *S. natans* subsp. *sulfidivorans* according to the List of Prokaryotic names with Standing in Nomenclature (LPSN)*.* The genus *Leptothrix* is characterized by its ability to oxidize manganese which is not observed in the genus *Sphaerotilus* (van Veen et al. 1978), excluding *S. mobilis* (Spring et al. 1996). *L. mobilis* was reclassified to the genus *Sphaerotilus* as *S. mobilis* in 2022 based on the genus boundary values of ANI (78.95–82.14%) and AAI (67.12–71.55%) proposed for the *Sphaerotilus-Leptothrix* group and related genera (Liu et al. 2022). The strains belonging to the non-validly described species *“Leptothrix discophora*” were classified as *Leptothrix cholodnii* and *L. discophora* in 1996 (Spring et al. 1996). After classification, the genus *Leptothrix* comprises four species: *Leptothrix ochracea*, *L. cholodnii*, *L. discophora*, and *Leptothrix lopholea*. The type strains of *L. ochracea*, *L. lopholea,* and *L. cholodnii* are not available from culture collections (Spring et al. 1996; Yarza et al. 2013), although *L. ochracea* is the type species of the genus *Leptothrix* (Skerman et al. 1980). Instead of the type strain, a reference strain of *L. cholodnii* was proposed and is available (Spring et al. 1996). No genomic data is available for the type strains of *L. ochracea*, *L. lopholea*, and *L. cholodnii* in the database.

In this study, we report the isolation and taxonomic characterization of a bacterial strain originating from the activated sludge of the sewage treatment plant in Yokohama exhibiting a sheathed morphology typical of the genus *Sphaerotilus*. Here, we describe the isolation of strain FB-5^ T^, a member of the genus *Sphaerotilus*, and propose that the isolate belongs to a novel species, *S. microaerophilus* sp. nov.

## Materials and methods

### Isolation and cultivation

Strain FB-5^ T^ was isolated from the activated sludge of the sewage treatment plant in Yokohama, Japan. The activated sludge was suspended with water and the suspension was streaked on an agar medium (named Screening medium) followed by incubation at 25 °C for 3 days. Colony isolation was performed three times at three-day intervals using the same medium at 25 °C. Screening medium was composed of 15 g/L agar, 0.15 g/L soluble starch, 50 mg/L (NH_4_)_2_SO_4_, 50 mg/L K_2_HPO_4_, 50 mg/L MgSO_4_∙7H_2_O, 50 mg/L KCl, 100 mg/L CaCO_3_, 14 mg/L Ca(NO_3_)_2_∙4H_2_O, 3 mg/L FeSO_4_∙7H_2_O, 0.3 mg/L H_3_BO_3_, 0.2 mg/L CoCl_2_∙6H_2_O, 0.1 mg/L ZnSO_4_∙7H_2_O, 0.03 mg/L MnCl_2_∙4H_2_O, 0.03 mg/L Na_2_MoO_4_∙2H_2_O, 0.02 mg/L NiCl_2_, 0.2 mg/L CuCl_2_, 0.4 mg/L thiamin HCl, and 0.01 mg/L vitamin B_12_. Colonies with a rough (hairy) appearance, possibly attributable to filamentous growth, were selected using this medium. For maintains, the isolate (strain FB-5^ T^) was subcultured on an agar plate (named APP medium) composed of 15 g/L agar, 2 g/L Proteose-Peptone No. 3 (Difco), and 0.01 mg/L vitamin B_12_ at 15–25 °C in the range of one week. For taxonomic characterization, the strain was statically cultured at 25 °C for 5 days in a medium (named GPPY medium) composed of 4 g/L glucose, 2 g/L Proteose-Peptone No. 3 (Difco), 0.2 g/L yeast extract (Difco), 0.2 g/L MgSO_4_‧7H_2_O, and 0.01 mg/L vitamin B_12_. For short-term storage in the range of one month, subcultures were performed at 15 °C using a semi-solidified GPPY medium containing 5 g/L agar. For long-term storage, the cells statically grown in GPPY medium at 25 °C for 5 days was stored at −80 °C in the presence of 20% glycerol. Lyophilization was not performed because of a loss of viability.

### Morphology

The morphology was examined using a JSM-7001F scanning electron microscope (SEM, JEOL, Tokyo, Japan), a JEM-2100F transmission electron microscope (TEM, JEOL, Tokyo, Japan), and a SPA-400/SPI3800N scanning probe microscope (SPM, Hitachi High-Technologies, Tokyo, Japan).

### Phenotypic analyses

Unless otherwise described, the cells statically grown in GPPY medium at 25 °C for 5 days was used for phenotypic characterization. Gram-staining was performed using Favor G Nissui (Nissui Pharmaceutical Co., Ltd., Tokyo, Japan) according to the supplier’s instructions. The utilization of organic compounds as sole carbon and energy sources was determined by monitoring the increase in turbidity (absorbance at 660 nm) after triplicate subcultures in a mineral medium (named NCM medium), which was used for the characterization of the *Sphaerotilus* strains (Gridneva et al.^2011^), mainly composed of (NH_4_)_2_SO_4_, CaCl_2_, MgSO_4_‧7H_2_O, Na_2_S_2_O_3_, and organic compound to be tested. Sulfur-dependent lithotrophic growth was examined using batch cultures under the conditions reported by Gridneva and collaborators (Gridneva et al.^2011^). Sulfur-dependent lithotrophic growth was also examined using fed-batch cultures feeding with Na_2_S solution (1 g/L) in a 500 mL-flask (four-necked round-bottom flask containing 300 mL of medium) at 25 °C using a modified DSMZ573 medium (named m-DSMZ573 medium) under the following conditions: medium, DSMZ573 medium (without sodium acetate) supplemented with 1 mL/L Wolfe’s vitamin solution (ATCC 2094 AN 1 medium); aeration, 0.1 L/min without agitation; pH, maintained at 7.2–7.8 using a pH controller (NPH-690D, Nisshin Rika, Tokyo, Japan); Na_2_S feeding, H_2_S concentration in the exhaust was maintained at 0.2–1 ppm using a H_2_S sensor (FECS50-100, Figaro Engineering, Osaka, Japan) in combination with a control device composed of E5CD and G3NA-205B-UTU (OMRON, Kyoto, Japan). In the fed-batch culture, bacterial H_2_S consumption (removal) was calculated based on the amount of Na_2_S solution added to the flask to maintain the H_2_S concentration in the exhaust. Growth was detected as relative light units (RLU) by the ATP (including ADP and AMP) bioluminescence assay using a Lumitester Smart (Kikkoman, Tokyo, Japan). Manganese oxidation was examined in a stab culture (25 °C, one month) using MSVP agar (Emerson and Ghiorse 1992; Siering and Ghiorse 1996; Takeda et al. 2002) containing 1 mM Mn^2+^. *L. cholodnii* ATCC 51168 (= SP-6) and *S. natans* JCM 20382 (= ATCC 15291) were used as the positive and negative controls, respectively. Note that the subspecies affiliation of *S. natans* JCM 20382 is not determined (Gridneva et al. 2011). Although these strains are not the type strains, they are commonly used as references to compare their phenotypic properties with those of related isolates (Sawayama et al. 2011; Nott et al. 2020; Kashiwabara et al. 2021; Kunoh et al. 2021). Growth under aerobic conditions was examined on GPPY agar (GPPY medium solidified with 1.5% agar) and APP medium at 25 °C for 10 days. Growth under microaerophilic conditions was examined by semi-solid-state cultivation in a test tube with a diameter of 1.8 cm at 25 °C for 10 days using GPPY medium supplemented with 0.5% agar. Microaerophilic growth was also examined on GPPY agar (GPPY medium solidified with 1.5% agar) at 25 °C for 10 days using AnaeroPouch-MicroAero (Mitsubishi Gas Chemical, Tokyo, Japan). Likewise, anaerobic growth was examined using AnaeroPouch-Anaero (Mitsubishi Gas Chemical), respectively. Catalase and oxidase activities were evaluated using 3% (v/v) H_2_O_2_ and 1% (w/v) tetramethyl-*p*-phenylenediamine, respectively, as previously described (Smibert 1994). The following characteristics were determined using the ID test NF-18 (Nissui Pharmaceutical): nitrate reduction to nitrite; nitrite reduction to nitrogen; gelatin liquefaction; indole production; hydrolysis of esculin, urea, and arginine; decarboxylation of lysine and ornithine; and β-galactosidase activity. The effect of temperature on growth was investigated using GPPY medium at 5–50 °C (5 °C intervals). The effect of pH was investigated at 25 °C using GPPY medium at pH 3–10 (1 pH unit intervals) adjusted with HCl or NaOH. The effect of NaCl concentration was investigated at 25 °C using GPPY medium supplemented with 0, 0.05, 0.1, 0.2, 0.3, 0.4, 0.5, 1, 2, or 3% (w/v) NaCl. The growth in static cultures was checked by monitoring turbidity (absorbance at 660 nm) for 5 days.

### Chemotaxonomic analysis

The cellular fatty acid composition was determined using the Sherlock Microbial Identification System (MIDI) according to the manufacturer’s instructions. The major respiratory quinones were determined using the method described by Tamaoka and collaborators (Tamaoka et al. 1983). PHB accumulation was determined using gas chromatography, as described previously (Takeda et al. 1995). For these experiments, cells grown in GPPY medium were used because strain FB-5^ T^ did not grow in the medium used for chemotaxonomic characterization of the closely related *Sphaerotilus* strains (Gridneva et al. 2011; Grabovich et al. 2021).

### 16S rRNA gene phylogeny

The 16S rRNA gene was amplified using colony PCR with the primers 27F (Jensen et al. 2009) and 1500R (Weisburg et al. 1991) and followed by Sanger sequencing (Applied Biosystems 3730xl DNA Analyzer; Applied Biosystems, Waltham, MA, USA) in two directions. The sequence was confirmed to be the same as that extracted from the genome assembly and deposited in DDBJ/ENA/GenBank under the accession number LC775240. Phylogenetic analysis using neighbor joining (NJ), unweighted pair group method with arithmetic mean (UPGMA), maximum likelihood (ML), and maximum parsimonious (MP) methods was performed based on the 16S rRNA gene sequences. Genes for analysis were extracted from the genome sequences (Table S1) of the reference strains using Barrnap 0.9 (https://github.com/tseemann/barrnap), followed by multiple alignment (Q-INS-i algorithm) using MAFFT version 7 (Katoh et al. 2019). NJ and UPGMA trees were constructed using PHYLIP 3.698 (https://evolution.genetics.washington.edu/phylip.html) based on pairwise sequence similarities (Table S2). Similarities were calculated using blastn implemented in BLAST 2.14.0 + . MP tree was constructed using MEGA 11.0.13 (Tamura et al. 2021). For a reliable estimation, only sequences within the *Sphaerotilus-Leptothrix* group were used, and the root was selected using the midpoint rooting method. The topological robustness of the NJ, UPGMA, and MP trees was evaluated using a bootstrap analysis with 1000 replicates. The maximum likelihood tree was constructed using IQ-TREE 2.3.3 (Minh et al. 2020) under the TN + F + I + G4 as the best-fit nucleotide substitution model, which was selected by the ModelFinder (Kalyaanamoorthy et al. 2017) according to the Bayesian information criterion. Branching robustness was estimated using the SH-like approximate likelihood ratio test (SH-aLRT) with 1000 replicates.

**Fig. 1:**
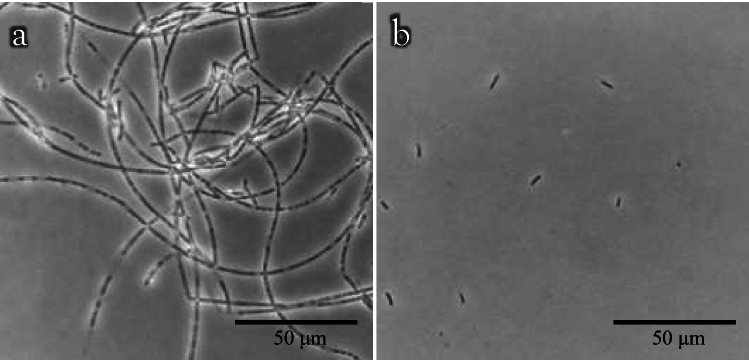
Phase-contrast microscopy images of strain FB-5^ T^. Strain FB-5^ T^ was statically cultivated at 25 °C for 4 days using GPPY medium. The aggregated cells were enclosed within a sheath (**a**) whereas non-aggregated cells remained without a sheath (**b**)

### Genome sequencing and analysis

The genome sequence of strain FB-5^ T^ was determined using the following procedure: after freezing in liquid nitrogen, the cell pellet was ground, and DNA was extracted using the Wizard Genomic DNA Purification Kit (Promega, Madison, CA, USA). After purification using AMPure XP (Beckman Coulter, Brea, CA, USA) and a DNeasy Power Clean Pro Cleanup Kit (Qiagen, Venlo, Netherlands), a DNA library was constructed using the SMRTbell Express Template Prep Kit 2.0 (Pacific Biosciences, Menlo Park, CA, USA). The library was sequenced using Sequel IIe (Pacific Biosciences) in combination with the Binding Kit 2.2 (Pacific Biosciences). Based on these sequences, consensus sequences were prepared and HiFi reads were obtained by omitting sequences of low reliability (< QC 20) using SMRT Link v11 software (Pacific Biosciences). After removing short reads (< 1000 bp) using Filtlong (https://github.com/rrwick/Filtlong), HiFi reads were assembled using Flye (https://github.com/fenderglass/Flye/blob/flye/docs/USAGE.md), and accuracy was confirmed using Bandage (Wick et al. 2015) and CheckM (Parks et al. 2015). Complete genomic data, including the plasmid, were deposited in DDBJ/ENA/GenBank under accession numbers AP025730 (genome) and AP025731 (plasmid). The assembled sequences were annotated using DFAST (Tanizawa et al. 2016), and 5311 genes were identified. The basic features of the genomic data used in this study, including those of strain FB-5^ T^, are listed in Table S1. The contamination and completeness were estimated using CheckM2 (Chklovski et al. 2023). The dDDH value was calculated using GGDC 3.0 (Meier-Kolthoff et al. 2022) with formular 2. A heat-map style matrix was produced using TBtools 2.016 (Chen et al. 2020). Multilocus sequence analysis based on the core gene set (concatenated sequences of 92 core genes from 32 genomes) was performed using UBCG 3.0 (Na et al. 2018), and a phylogenetic tree was constructed using FastTree (Price et al. 2010) implemented in the UBCG pipeline. Branching robustness was estimated using SH-aLRT with 1000 replicates. The genome-to-genome distance was calculated by AAI using EzAAI 1.2.2 (Kim et al. 2021), and the AAI matrix was produced using TBtools 2.016. The corresponding matrix plot was generated using PHYLIP 3.698. The genome-to-genome distance was further estimated by ANI using FastANI 1.33 (Jain et al. 2018). The ANI matrix was produced using TBtools 2.016, and the corresponding matrix plot was produced using PHYLIP 3.698.

## Results and discussion

### Isolation and maintenance

Initially, we attempted to isolate strains of the genus *Haliscomenobacter* (a genus in the phylum *Bacteroidota* comprising sheath-forming filamentous bacteria) capable of degrading various macromolecules, including starch and hyaluronate (Mori et al. 2023), using Screening medium containing starch. After repeated streak purification, a filamentous strain was isolated as a single colony and designated as strain FB-5^ T^. The colonies of strain FB-5^ T^ on Screening medium exhibited a loss of viability after 7 days of cultivation. Growth was observed on a starch-free Screening medium. No growth was observed in agar-free (liquid) Screening medium. These results suggested that the growth of the isolate was supported by organic impurities in the agar and that strain FB-5^ T^ was not a member of the genus *Haliscomenobacter*. The colonies formed on APP medium were irregularly (rough) shaped (Fig. S1a). Smooth colonies were rarely observed as well (Fig. S1b). Colonies of either shape produced colonies of both shapes in subculture. A rough colony was used for taxonomic characterization and subculture.

### Morphology

**Fig. 2:**
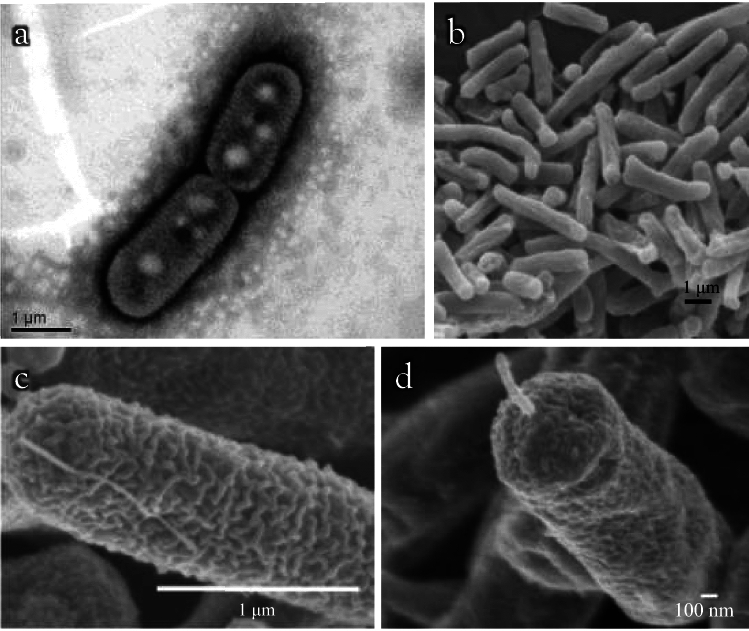
Transmission (**a**) and scanning (**b-d**) electron microscopy images of strain FB-5^ T^. The cells grown on GPPY medium were negatively stained with phosphotungstic acid and subjected to transmission electron microscopy (**a**). The cells for scanning electron microscopy (**b-d**) were fixed with glutaraldehyde and osmium tetraoxide. Coating was performed with osmium

Using phase-contrast microscopy, both the filaments (lines of cells enclosed by sheaths; Fig. 1a) and single rod-shaped cells (cells without a sheath; Fig. 1b) were observed in static cultures using GPPY medium. Single rod-shaped cells occasionally exhibited motility. Cells were not connected, and filamentation was caused by sheath formation. Filamentation (sheath formation) was promoted by the addition of 0.1 g/L CaCO_3_ to the medium. The enhancement of sheath formation by Ca^2+^ and Mg^2+^ has been observed in *L. cholodnii* (Kunoh et al. 2021). However, no effect of Mg^2+^ for sheath formation of strain FB-5^ T^ was suggested, as similar filamentation was observed in GPPY medium with or without MgSO_4_∙7H_2_O. Sheath formation was confirmed by SPM observation (Fig. S2). Filaments were commonly observed in the rough colonies (Fig. S1a) formed on a solid medium. In contrast, filaments were rare in the smooth colonies (Fig. S1b). Accordingly, a rough colony was selected to maintain the sheath-forming ability. Sheaths were not detected using TEM (Fig. 2a) or SEM (Fig. 2b), suggesting a weak sheath-forming capability of strain FB-5^ T^. The electron microscopy images (Fig. 2) revealed a cell size of 0.7–1.0 µm in width and 2.0–6.5 µm in length. The *sthA* (AB050638) and *lthB* (ACB33244) genes are essential glycosyl transferase genes for sheath formation in *S. natans* and *L. cholodnii*, respectively (Suzuki et al. 2002; Kunoh et al. 2023). A homology search using BLAST showed a *sthA*-like gene (WP_251972251/BDI04101) with 78% identity in the genome of strain FB-5^ T^, whereas a *lthB*-like gene was not detected, suggesting that strain FB-5^ T^ forms a *Sphaerotilus*-type sheath (Kondo et al. 2011; Kashiwabara et al. 2021). *Sphaerotilus*-type sheath is mainly composed of  glucuronic acid, galactosamine and glucose (Kondo 2011; Kashiwabara et al. 2021). In contrast, *Leptothrix*-type sheath contains *N*-acetylgalactose, galactosamine, galacturonic acid, and glucosamine (Takeda et al. 2005). Purification of the sheath and subsequent sugar composition analysis are required to determine the type of sheath of strain FB-5^ T^, which will be our future study. Traces of a single polar flagellum (the remaining short flagellum) were rarely observed in the SEM images (Fig. 2c, d). We assume that the motility of strain FB-5^ T^ is attributed to a single polar flagellum, as described for other members of the genera *Sphaerotilus* and *Leptothrix* (Spring et al. 1996; Grabovich et al. 2021).

### Physiology

Strain FB-5^ T^ was stained Gram-negative. Growth was observed in GPPY medium containing 0–0.5% NaCl but not observed in the presence of 1–3% NaCl. Growth was observed only in media containing amino acid or amino acid-based organic compounds such as peptone, Proteose-Peptone No. 3, tryptone, yeast extract, and casamino acids (Table S3). To the best of our knowledge, Proteose-Peptone No. 3 is the most suitable energy and carbon source for growth. No sugars or organic acids served as the sole growth substrate. The preference for peptides and amino acids is a distinguishing feature of strain FB-5^ T^ in comparison to other members of the genus *Sphaerotilus*. Vitamin B_12_ enhanced the growth of the strain because its growth was poor when vitamin B_12_ was omitted from GPPY medium. Vitamin B_12_ requirements are commonly recognized in the *Sphaerotilus* and *Leptothrix* strains (Okrend and Dondero 1964; Emerson and Ghiorse 1992). Sulfur-dependent lithotrophic growth was not observed in batch culture; however, growth (increase in RLU) and consumption of H_2_S were detected in fed-batch culture. Continuous supply of sulfide is probably desirable for lithotrophic cultivation of strain FB-5^ T^. As shown in Fig. S3, manganese oxidation was not observed in strain FB-5^ T^. Poor growth was observed in the uppermost part of the stab culture of strain FB-5^ T^, suggesting the strain is facultatively microaerophilic and prefers microaerophilic conditions rather than aerobic conditions, unlike *S. natans* and *L. cholodnii*. *S. natans* has been reported to oxidize iron, coupled with nitrate reduction catalyzed by nitrate reductase (WP_037485935) (Park et al. 2014). The amino acid sequence of the nitrate reductase of *S. natans* showed 71% identity with a putative nitrate reductase (WP_251972978) of strain FB-5^ T^ in a BLAST search, suggesting that the strain has iron-oxidizing potential. To confirm the preference of strain FB-5^ T^ for growth under microaerophilic conditions, a semi-solid culture was performed. Poor growth was observed at the air-medium interface, whereas colonies formed 0.3–1 cm below the interface different from *S. natans* (Fig. S4), indicating that strain FB-5^ T^ is microaerophilic. No growth was observed under microaerophilic and anaerobic conditions produced by AnaeroPouch-MicroAero and AnaeroPouch-Anaero, respectively. Since AnaeroPouch-MicroAero produces atmosphere of 6–12% O_2_, strain FB-5^ T^ is expected to prefer O_2_ concentrations above 12% and less than 21%. Additionally, strain FB-5^ T^ was cytochrome oxidase-positive same as the *Sphaerotilus* strains, but catalase-negative different from the *Sphaerotilus* strains. Nitrate was reduced to nitrite. Nitrite was not reduced to nitrogen. Gelatin was not liquefied. Indole was not produced. Esculin, urea, and arginine were not hydrolyzed. Lysine and ornithine were not decarboxylated. β-Galactosidase activity was negative. Growth was observed in a temperature range of 15–35 °C, with 30 °C being optimal. The optimum pH was 7.5, whereas growth was observed in the pH range of 7–8.

### Chemotaxonomy

As shown in Table S4, the fatty acids detected from strain FB-5^ T^ were C_16:1_ω7 (49.8%), C_16:0_ (25.4%), C_12:0_ (10.8%), C_18:1_ω7 (5.5%), and C_10:0_ 3-hydroxy (1.4%). Although the content of C_12:0_ was relatively high, the overall fatty acid composition was similar to that of other *Sphaerotilus* strains, including *S. mobilis* (Spring et al. 1996; Gridneva et al. 2011). The major respiratory quinone in strain FB-5^ T^ was UQ-8. The accumulation of PHB was confirmed. UQ-8 and PHB are commonly detected in the genus *Sphaerotilus* (Grabovich et al. 2021).

### 16S rRNA gene-based phylogeny

**Fig. 3:**
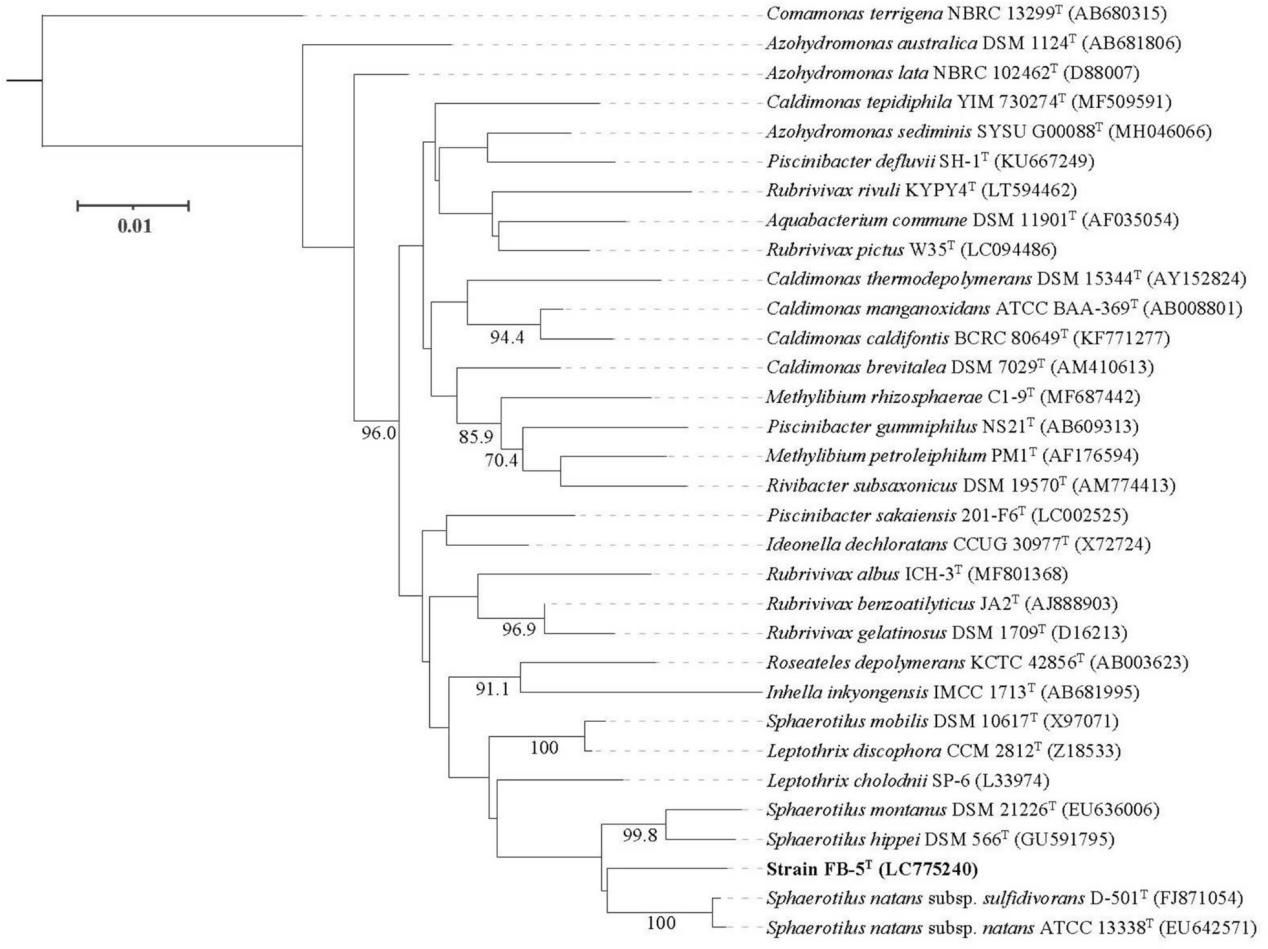
Phylogenetic tree (NJ) based on 16S rRNA gene sequences showing the relationship between strain FB-5^ T^ and related strains. The percentages (> 80%) for 1000 bootstraps are shown next to the branches. The scale represents 0.01 nucleotide substitutions per site

The pairwise identities of the 16S rRNA gene sequences of strains related to strain FB-5^ T^ are listed in Table S2. As shown in the phylogenetic tree (NJ) based on the 16S rRNA gene (Fig. 3), strain FB-5^ T^ was closely related to strains of the genus *Sphaerotilus*, particularly to *S. natans* subsup. *sulfidivorans* (98.0% similarity) followed by *S. natans* subsup. *natans* (97.8% similarity). Phylogenetic analysis using ML, UPGMA, and MP methods supported this result. The level of identity with *S. hippei* was 97.8%, with *S. montanus* 97.2%, and with *S. mobilis* 96.5%.

### Genomic features

The genome sequence of strain FB-5^ T^ determined in this study exhibited a low level of contamination (0.26%), with 99.99% completeness, as shown in Table S1. The genomic DNA G + C content of strain FB-5^ T^ was 69.16%, which was within the range (68.16 − 70.03%) of the *Sphaerotilus* strains. In the genome, 5,236 protein-coding and 75 non-coding genes were identified. The genome size of strain FB-5^ T^ (6.06 Mbp) was higher than that of the *Sphaerotilus* strains (4.39 − 5.07 Mbp), which distinguished the isolate from related strains of the genus *Sphaerotilus*. Supporting the lithotrophic growth capability of strain FB-5^ T^ in fed-batch culture, the genes required for sulfur metabolism and Calvin–Benson–Bassham cycle were observed in the genome as listed in Tables S5 and S6, respectively. Accordingly, strain FB-5^ T^ was revealed to be lithotrophic, the same as *S. natans subsp. sulfidivorans* (Gridneva et al. 2011; Grabovich et al. 2021). Comparative genomic analysis was performed to further characterize the strain as a member of the *Sphaerotilus-Leptothrix* group.

**Fig. 4:**
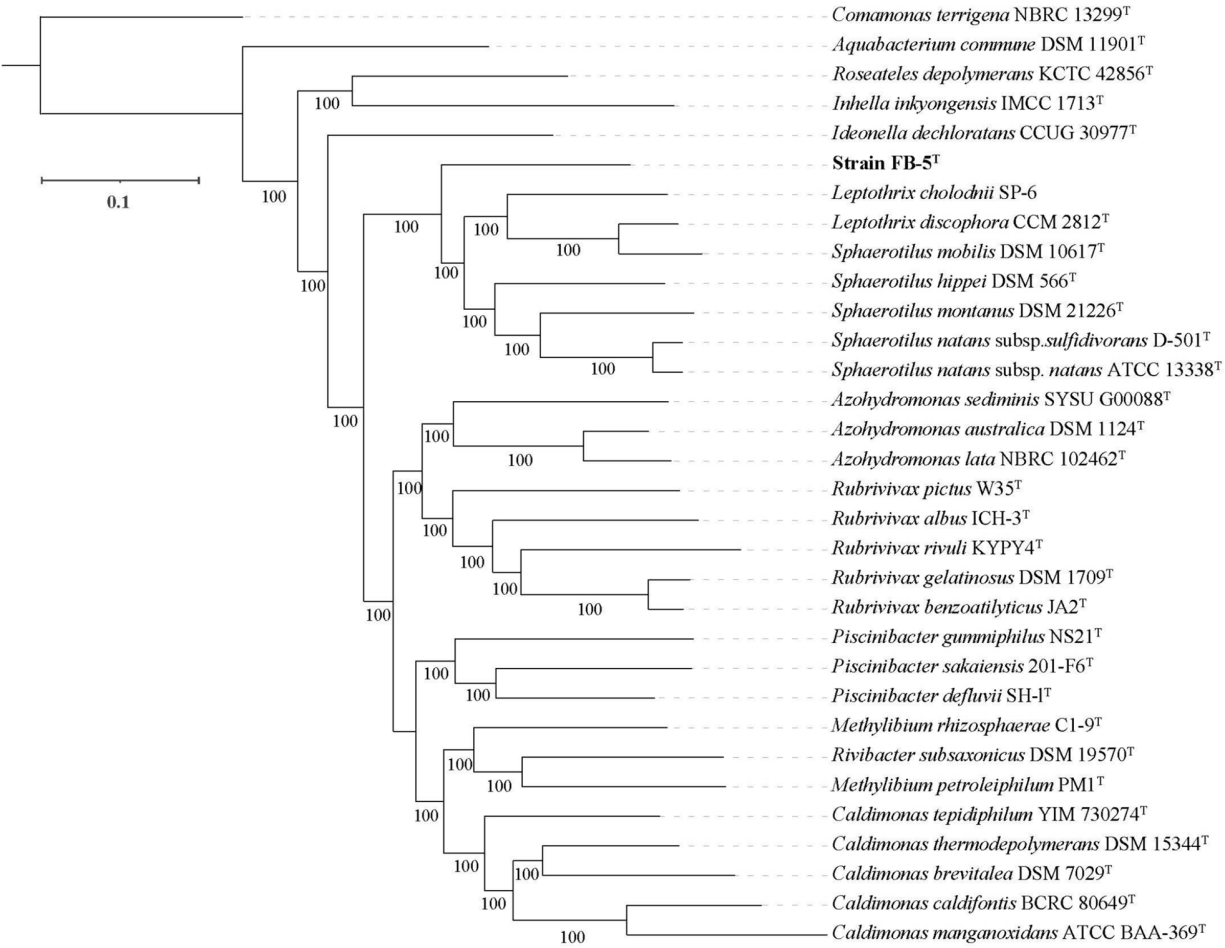
Phylogenetic tree (ML) based on core genome sequences showing the relationship between strain FB-5^ T^ and related strains. The percentages (> 70%) for 1000 bootstraps are shown next to the branches. The scale represents 0.1 nucleotide substitutions per site

The dDDH values between strain FB-5^ T^ and the strains of the genus *Sphaerotilus* ranged from 21.8 to 22.6% as shown in the heat map style matrix (Fig. S5). The values were much lower than the species cutoff value of 70% (Goris et al. 2007; Meier-Kolthoff et al. 2013), indicating that strain FB-5^ T^ represents a new species in the genus *Sphaerotilus*. In the phylogenetic tree constructed based on the core genes set (Fig. 4), strain FB-5^ T^ was located outside the clade formed by other members of the *Sphaerotilus-Leptothrix* group.

The AAI values against the species in the *Sphaerotilus-Leptothrix* group ranged from 71.0 to 72.5% (Fig. S6), with the lowest value (71.0%) against *S. natans* subsp. *natans* and the highest value (72.5%) against *L. cholodnii.* In the AAI matrix plot (Fig. S7), strain FB-5^ T^ formed a clade with *L. cholodnii*, *L. discophora*, and *S. mobilis*, suggesting that it was closely related to these species. A neighboring clade was formed by the *Sphaerotilus* strains other than *S. mobilis*. AAI values of *L. cholodnii* and *L. discophora* against the *Sphaerotilus* strains, including strain FB-5^ T^ ranged from 70.7 to 72.6% (Fig. S6). Considering the AAI relatedness and *Sphaerotilus-Leptothrix* group-specific genus boundary AAI value of 67.12–71.55% (Liu et al. 2022), strain FB-5^ T^ should be classified into the genus *Sphaerotilus*. The ANI values between strain FB-5^ T^ and the strains of the *Sphaerotilus-Leptothrix* group ranged from 81.7 to 82.5% (Fig. S8). In the ANI matrix plot (Fig. S9), strain FB-5^ T^ was located distantly within the clades of the genera *Sphaerotilus* and *Leptothrix*, suggesting its novelty. Based on the *Sphaerotilus*-*Leptothrix* group-specific genus boundary ANI value of 78.95–82.14% (Liu et al. 2022), strain FB-5^ T^ should be classified into the genus *Sphaerotilus*.

## Conclusion

Table 1 summarizes the major phenotypic features of strain FB-5^ T^ that distinguish it from known closely related species. Because strain FB-5^ T^ did not oxidize manganese, it could be easily distinguished from *S. mobilis* and strains of the genus *Leptothrix*. The cell shape and size were almost identical to those of the known species of the genus *Sphaerotilus*. Sheath formation by strain FB-5^ T^ was not particularly stable in the absence of calcium, which differs from that observed in other known species of the genus *Sphaerotilus*. Strain FB-5^ T^ is microaerophilic and catalase-negative, whereas the type strains of the genus *Sphaerotilus* are strictly aerobic and catalase-positive. Because lithotrophic sulfur oxidation is possible, strain FB-5^ T^ is the second lithotrophic member found after *S. natans* subsp. *sulfidivorans* in the genus *Sphaerotilus*. The inability of strain FB-5^ T^ to utilize sugars and organic acids as the sole energy and carbon sources distinguished it from known species in the genus *Sphaerotilus*. Based on the differences in phenotypic properties and genomic features, this strain should be classified as the type strain of a new species, *Sphaerotilus microaerophilus* sp. nov.

**Table 1:** Distinguishing characteristics of strain FB-5^ T^ and related type strains

Characteristics	Strain FB-5^ T^	*S. natans* subsp. *natans* DSM 6575^ Ta^	*S. natans.* subsp. *sulfidivorans* D-501^ Ta^	*S. hippiei* DSM 566^ Ta^	*S. montanus* HS^Ta^	*S. mobilis* Fox-1^ Ta^	*L. discophora* LMG 8141^ Ta^	*L. cholodnii* LMG 7171^ Ta^	
Cell size (μm) width length	0.7–1.0 2.0–6.5	1.2–2.0 2.0–6.0	1.0–2.0 3.9–6.0	0.7–1.5 2.0–6.2	0.8–1.4 3.3–6.0	0.6–0.8 1.5–12	0.6–0.8 2.5–12	0.7–1.5 2.5–15	
Sheath formation	± ^b^	+ ^b^	+	+	+	− ^b^	±	±	
Microaerophilic	+	−	−	−	−	−	−	−	
Catalase	−	+	+	+	+	ND^c^	ND	ND	
Manganese oxidation	−	−	−	−	−	+	+	+	
Sulfide oxidation	+	−	+	−	−	−	−	−	
Growth on glucose	−	+	+	+	+	−	−	−	
fumarate	−	+ poor growth	−	−	+	+	−	−	
proline	−	+	+	+	+	−	+	+	
pyruvate	−	+	+	+	+	−	−	−	
Genome size (Mbp)^d^	6.06	4.63	4.39	4.43	5.07	4.65	4.65	ND	
G + C content (%)^e^	69.16	69.94	69.88	70.03	68.16	69.00	70.12	70	

^a^Data from Grabovich et al. (2021), Gridneva et al. (2011), and Spring et al. (1996)

^b^+ Positive or supported growth;− negative or did not support growth; ± sheath formation was easily lost during maintenance

^c^Not determined

^d^No genomic data is available for *L. cholodnii* LMG 7171^ T^

^e^Based on genome-wide sequences other than *L. cholodnii* LMG 7171^ T^, which (mol%) was determined by the thermal denaturation method (Spring et al. 1996)

## Description of *Sphaerotilus microaerophilus* sp. nov.

*Sphaerotilus microaerophilus* (mi.cro. a.e.ro'phi.lus. Gr. masc. adj. *mikros*, small; Gr. masc. n. *aêr*, air; N.L. masc. adj. *philus* (from Gr. masc. adj. *philos*), friend; N.L. masc. adj. *microaerophilus*, loving conditions of low air, referring to the low oxygen preference of the type strain, FB-5).

Straight rod-shaped cells with rounded ends are 0.7–1.0 × 2.0–6.5 µm in size, motile by means of a single polar flagellum. A few cells are enclosed within the sheaths. The sheath-forming ability is unstable in the absence of calcium salts. The rough colonies with fibrous edges are mostly of sheath-forming cells, whereas the smooth colonies are mostly sheathless. Facultatively microaerophilic. Semi-solidified GPPY medium is suitable for growth. Colonies are colorless. The temperature range for growth is 15–35 °C, with 30 °C being optimal. The pH range for growth is 7–8, with an optimal pH of 7.5. Aspartate, glutamate, methionine, tyrosine, peptone, yeast extract, tryptone, casamino acids, and Proteose-Peptone No. 3 are utilized in NCM medium as sole carbon sources. Ethanol, butanol, iso-butanol, propanol, glucose, glycerol, sorbitol, sorbose, arabinose, fructose, lactose, galactose, mannose, maltose, sucrose, raffinose, acetate, formate, citrate, lactate, malate, malonate, pyruvate, benzoate, oxalate, oxaloacetate, 2-oxoglutarate, succinate, fumarate, glycolate, aconitate, alanine, arginine, asparagine, cysteine, glutamine, glycine, histidine, isoleucine, leucine, lysine, phenylalanine, proline, serine, threonine, tryptophan, and valine are not utilized in NCM medium as sole carbon sources. Lithotrophic sulfur oxidation occurs in m-DSMZ573 medium with continues feeding of sulfide. Oxidase positive. Catalase negative. Nitrate is reduced to nitrite (ID test NF-18). Gelatin is not liquefied and indole is not formed (ID test NF-18). Negative for esculin hydrolysis, arginine hydrolysis, urea degradation, lysine decarboxylation, ornithine decarboxylation, and β-galactosidase (ID test NF-18). No growth in GPPY medium supplemented with 3% (w/v) NaCl. The major quinone is UQ-8. The major fatty acids are C_16:1_ω7, C_16:0_, and C_12:0_. The genomic DNA G + C content is 69.16%. The type strain is FB-5^ T^ (= JCM 35424^ T^ = KACC 23146^ T^) isolated from the activated sludge of a sewage treatment plant in Yokohama, Japan. The GenBank accession numbers for the 16S rRNA gene and the genome of the type strain are LC775240 and AP025730, respectively.

### Supplementary Information

Below is the link to the electronic supplementary material.

Supplementary file1 (PPTX 6790 KB)

## Data Availability

The near-complete 16S rRNA gene sequence of strain FB-5^ T^ can be obtained in GenBank/EMBL/DDBJ accession number LC775240. The whole-genome data for strain FB-5^ T^ were deposited in DDBJ/ENA/GenBank under the accession numbers AP025730 (genome) and AP025731 (plasmid). Strain FB-5^ T^ was deposited in the Japan Collection of Microorganisms (JCM) and Korean Agricultural Culture Collection (KACC) as JCM 35424^ T^ and KACC 23146^ T^, respectively.

## Abbreviations

AAI: Average amino acid identity
ANI: Average nucleotide identity
dDDH: Digital DNA-DNA hybridization
PHB: Polyhydroxybutyrate
