# Supplementary material for: Taxonomic characterization of Sphaerotilus microaerophilus sp. nov., a sheath-forming microaerophilic bacterium of activated sludge origin
Source: Arch Microbiol. 2024 May 10;206(6):252. doi: 10.1007/s00203-024-03991-9 (PMC11087309; doi:10.1007/s00203-024-03991-9)
Supplement: Supplementary file 1 — Supplementary file1 (PPTX 6790 KB) [file 203_2024_3991_MOESM1_ESM.pptx]

## Slide 1
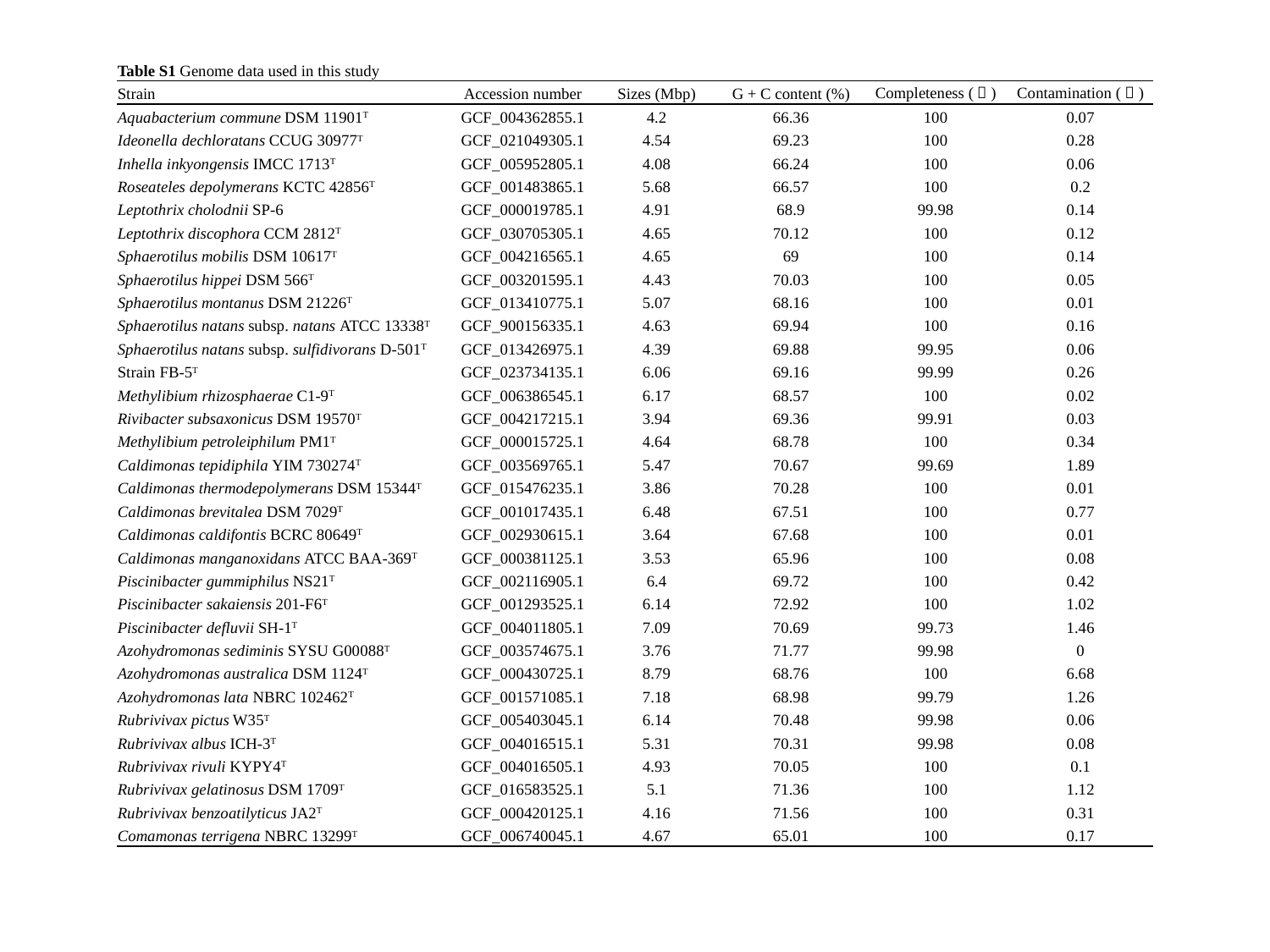

| Table S1 Genome data used in this study | | | | | |
| --- | --- | --- | --- | --- | --- |
| Strain | Accession number | Sizes (Mbp) | G + C content (%) | Completeness (％) | Contamination (％) |
| Aquabacterium commune DSM 11901T | GCF\_004362855.1 | 4.2 | 66.36 | 100 | 0.07 |
| Ideonella dechloratans CCUG 30977T | GCF\_021049305.1 | 4.54 | 69.23 | 100 | 0.28 |
| Inhella inkyongensis IMCC 1713T | GCF\_005952805.1 | 4.08 | 66.24 | 100 | 0.06 |
| Roseateles depolymerans KCTC 42856T | GCF\_001483865.1 | 5.68 | 66.57 | 100 | 0.2 |
| Leptothrix cholodnii SP-6 | GCF\_000019785.1 | 4.91 | 68.9 | 99.98 | 0.14 |
| Leptothrix discophora CCM 2812T | GCF\_030705305.1 | 4.65 | 70.12 | 100 | 0.12 |
| Sphaerotilus mobilis DSM 10617T | GCF\_004216565.1 | 4.65 | 69 | 100 | 0.14 |
| Sphaerotilus hippei DSM 566T | GCF\_003201595.1 | 4.43 | 70.03 | 100 | 0.05 |
| Sphaerotilus montanus DSM 21226T | GCF\_013410775.1 | 5.07 | 68.16 | 100 | 0.01 |
| Sphaerotilus natans subsp. natans ATCC 13338T | GCF\_900156335.1 | 4.63 | 69.94 | 100 | 0.16 |
| Sphaerotilus natans subsp. sulfidivorans D-501T | GCF\_013426975.1 | 4.39 | 69.88 | 99.95 | 0.06 |
| Strain FB-5T | GCF\_023734135.1 | 6.06 | 69.16 | 99.99 | 0.26 |
| Methylibium rhizosphaerae C1-9T | GCF\_006386545.1 | 6.17 | 68.57 | 100 | 0.02 |
| Rivibacter subsaxonicus DSM 19570T | GCF\_004217215.1 | 3.94 | 69.36 | 99.91 | 0.03 |
| Methylibium petroleiphilum PM1T | GCF\_000015725.1 | 4.64 | 68.78 | 100 | 0.34 |
| Caldimonas tepidiphila YIM 730274T | GCF\_003569765.1 | 5.47 | 70.67 | 99.69 | 1.89 |
| Caldimonas thermodepolymerans DSM 15344T | GCF\_015476235.1 | 3.86 | 70.28 | 100 | 0.01 |
| Caldimonas brevitalea DSM 7029T | GCF\_001017435.1 | 6.48 | 67.51 | 100 | 0.77 |
| Caldimonas caldifontis BCRC 80649T | GCF\_002930615.1 | 3.64 | 67.68 | 100 | 0.01 |
| Caldimonas manganoxidans ATCC BAA-369T | GCF\_000381125.1 | 3.53 | 65.96 | 100 | 0.08 |
| Piscinibacter gummiphilus NS21T | GCF\_002116905.1 | 6.4 | 69.72 | 100 | 0.42 |
| Piscinibacter sakaiensis 201-F6T | GCF\_001293525.1 | 6.14 | 72.92 | 100 | 1.02 |
| Piscinibacter defluvii SH-1T | GCF\_004011805.1 | 7.09 | 70.69 | 99.73 | 1.46 |
| Azohydromonas sediminis SYSU G00088T | GCF\_003574675.1 | 3.76 | 71.77 | 99.98 | 0 |
| Azohydromonas australica DSM 1124T | GCF\_000430725.1 | 8.79 | 68.76 | 100 | 6.68 |
| Azohydromonas lata NBRC 102462T | GCF\_001571085.1 | 7.18 | 68.98 | 99.79 | 1.26 |
| Rubrivivax pictus W35T | GCF\_005403045.1 | 6.14 | 70.48 | 99.98 | 0.06 |
| Rubrivivax albus ICH-3T | GCF\_004016515.1 | 5.31 | 70.31 | 99.98 | 0.08 |
| Rubrivivax rivuli KYPY4T | GCF\_004016505.1 | 4.93 | 70.05 | 100 | 0.1 |
| Rubrivivax gelatinosus DSM 1709T | GCF\_016583525.1 | 5.1 | 71.36 | 100 | 1.12 |
| Rubrivivax benzoatilyticus JA2T | GCF\_000420125.1 | 4.16 | 71.56 | 100 | 0.31 |
| Comamonas terrigena NBRC 13299T | GCF\_006740045.1 | 4.67 | 65.01 | 100 | 0.17 |

## Slide 2
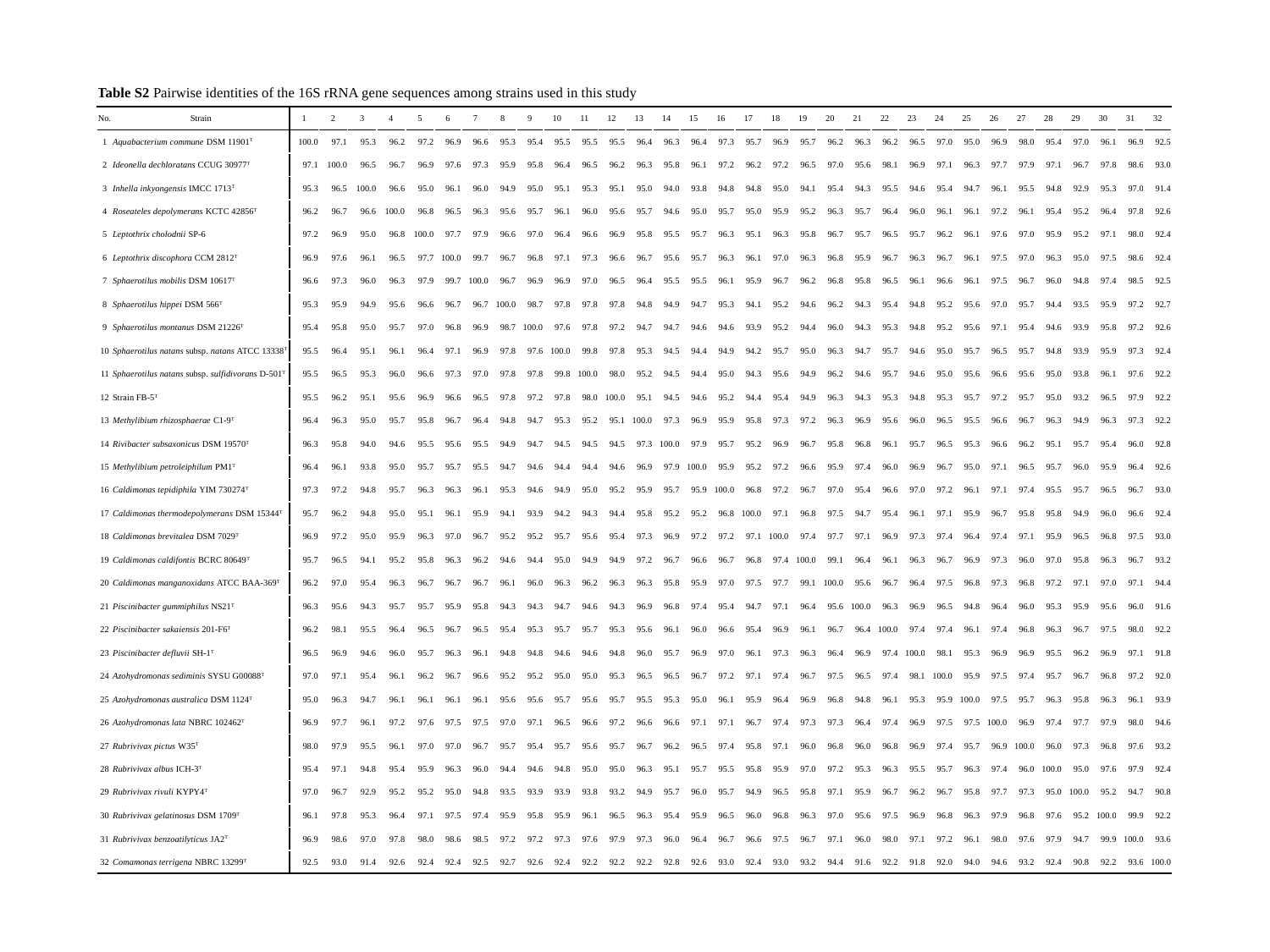

| Table S2 Pairwise identities of the 16S rRNA gene sequences among strains used in this study | | | | | | | | | | | | | | | | | | | | | | | | | | | | | | | | | |
| --- | --- | --- | --- | --- | --- | --- | --- | --- | --- | --- | --- | --- | --- | --- | --- | --- | --- | --- | --- | --- | --- | --- | --- | --- | --- | --- | --- | --- | --- | --- | --- | --- | --- |
| No. | Strain | 1 | 2 | 3 | 4 | 5 | 6 | 7 | 8 | 9 | 10 | 11 | 12 | 13 | 14 | 15 | 16 | 17 | 18 | 19 | 20 | 21 | 22 | 23 | 24 | 25 | 26 | 27 | 28 | 29 | 30 | 31 | 32 |
| 1 | Aquabacterium commune DSM 11901T | 100.0 | 97.1 | 95.3 | 96.2 | 97.2 | 96.9 | 96.6 | 95.3 | 95.4 | 95.5 | 95.5 | 95.5 | 96.4 | 96.3 | 96.4 | 97.3 | 95.7 | 96.9 | 95.7 | 96.2 | 96.3 | 96.2 | 96.5 | 97.0 | 95.0 | 96.9 | 98.0 | 95.4 | 97.0 | 96.1 | 96.9 | 92.5 |
| 2 | Ideonella dechloratans CCUG 30977T | 97.1 | 100.0 | 96.5 | 96.7 | 96.9 | 97.6 | 97.3 | 95.9 | 95.8 | 96.4 | 96.5 | 96.2 | 96.3 | 95.8 | 96.1 | 97.2 | 96.2 | 97.2 | 96.5 | 97.0 | 95.6 | 98.1 | 96.9 | 97.1 | 96.3 | 97.7 | 97.9 | 97.1 | 96.7 | 97.8 | 98.6 | 93.0 |
| 3 | Inhella inkyongensis IMCC 1713T | 95.3 | 96.5 | 100.0 | 96.6 | 95.0 | 96.1 | 96.0 | 94.9 | 95.0 | 95.1 | 95.3 | 95.1 | 95.0 | 94.0 | 93.8 | 94.8 | 94.8 | 95.0 | 94.1 | 95.4 | 94.3 | 95.5 | 94.6 | 95.4 | 94.7 | 96.1 | 95.5 | 94.8 | 92.9 | 95.3 | 97.0 | 91.4 |
| 4 | Roseateles depolymerans KCTC 42856T | 96.2 | 96.7 | 96.6 | 100.0 | 96.8 | 96.5 | 96.3 | 95.6 | 95.7 | 96.1 | 96.0 | 95.6 | 95.7 | 94.6 | 95.0 | 95.7 | 95.0 | 95.9 | 95.2 | 96.3 | 95.7 | 96.4 | 96.0 | 96.1 | 96.1 | 97.2 | 96.1 | 95.4 | 95.2 | 96.4 | 97.8 | 92.6 |
| 5 | Leptothrix cholodnii SP-6 | 97.2 | 96.9 | 95.0 | 96.8 | 100.0 | 97.7 | 97.9 | 96.6 | 97.0 | 96.4 | 96.6 | 96.9 | 95.8 | 95.5 | 95.7 | 96.3 | 95.1 | 96.3 | 95.8 | 96.7 | 95.7 | 96.5 | 95.7 | 96.2 | 96.1 | 97.6 | 97.0 | 95.9 | 95.2 | 97.1 | 98.0 | 92.4 |
| 6 | Leptothrix discophora CCM 2812T | 96.9 | 97.6 | 96.1 | 96.5 | 97.7 | 100.0 | 99.7 | 96.7 | 96.8 | 97.1 | 97.3 | 96.6 | 96.7 | 95.6 | 95.7 | 96.3 | 96.1 | 97.0 | 96.3 | 96.8 | 95.9 | 96.7 | 96.3 | 96.7 | 96.1 | 97.5 | 97.0 | 96.3 | 95.0 | 97.5 | 98.6 | 92.4 |
| 7 | Sphaerotilus mobilis DSM 10617T | 96.6 | 97.3 | 96.0 | 96.3 | 97.9 | 99.7 | 100.0 | 96.7 | 96.9 | 96.9 | 97.0 | 96.5 | 96.4 | 95.5 | 95.5 | 96.1 | 95.9 | 96.7 | 96.2 | 96.8 | 95.8 | 96.5 | 96.1 | 96.6 | 96.1 | 97.5 | 96.7 | 96.0 | 94.8 | 97.4 | 98.5 | 92.5 |
| 8 | Sphaerotilus hippei DSM 566T | 95.3 | 95.9 | 94.9 | 95.6 | 96.6 | 96.7 | 96.7 | 100.0 | 98.7 | 97.8 | 97.8 | 97.8 | 94.8 | 94.9 | 94.7 | 95.3 | 94.1 | 95.2 | 94.6 | 96.2 | 94.3 | 95.4 | 94.8 | 95.2 | 95.6 | 97.0 | 95.7 | 94.4 | 93.5 | 95.9 | 97.2 | 92.7 |
| 9 | Sphaerotilus montanus DSM 21226T | 95.4 | 95.8 | 95.0 | 95.7 | 97.0 | 96.8 | 96.9 | 98.7 | 100.0 | 97.6 | 97.8 | 97.2 | 94.7 | 94.7 | 94.6 | 94.6 | 93.9 | 95.2 | 94.4 | 96.0 | 94.3 | 95.3 | 94.8 | 95.2 | 95.6 | 97.1 | 95.4 | 94.6 | 93.9 | 95.8 | 97.2 | 92.6 |
| 10 | Sphaerotilus natans subsp. natans ATCC 13338T | 95.5 | 96.4 | 95.1 | 96.1 | 96.4 | 97.1 | 96.9 | 97.8 | 97.6 | 100.0 | 99.8 | 97.8 | 95.3 | 94.5 | 94.4 | 94.9 | 94.2 | 95.7 | 95.0 | 96.3 | 94.7 | 95.7 | 94.6 | 95.0 | 95.7 | 96.5 | 95.7 | 94.8 | 93.9 | 95.9 | 97.3 | 92.4 |
| 11 | Sphaerotilus natans subsp. sulfidivorans D-501T | 95.5 | 96.5 | 95.3 | 96.0 | 96.6 | 97.3 | 97.0 | 97.8 | 97.8 | 99.8 | 100.0 | 98.0 | 95.2 | 94.5 | 94.4 | 95.0 | 94.3 | 95.6 | 94.9 | 96.2 | 94.6 | 95.7 | 94.6 | 95.0 | 95.6 | 96.6 | 95.6 | 95.0 | 93.8 | 96.1 | 97.6 | 92.2 |
| 12 | Strain FB-5T | 95.5 | 96.2 | 95.1 | 95.6 | 96.9 | 96.6 | 96.5 | 97.8 | 97.2 | 97.8 | 98.0 | 100.0 | 95.1 | 94.5 | 94.6 | 95.2 | 94.4 | 95.4 | 94.9 | 96.3 | 94.3 | 95.3 | 94.8 | 95.3 | 95.7 | 97.2 | 95.7 | 95.0 | 93.2 | 96.5 | 97.9 | 92.2 |
| 13 | Methylibium rhizosphaerae C1-9T | 96.4 | 96.3 | 95.0 | 95.7 | 95.8 | 96.7 | 96.4 | 94.8 | 94.7 | 95.3 | 95.2 | 95.1 | 100.0 | 97.3 | 96.9 | 95.9 | 95.8 | 97.3 | 97.2 | 96.3 | 96.9 | 95.6 | 96.0 | 96.5 | 95.5 | 96.6 | 96.7 | 96.3 | 94.9 | 96.3 | 97.3 | 92.2 |
| 14 | Rivibacter subsaxonicus DSM 19570T | 96.3 | 95.8 | 94.0 | 94.6 | 95.5 | 95.6 | 95.5 | 94.9 | 94.7 | 94.5 | 94.5 | 94.5 | 97.3 | 100.0 | 97.9 | 95.7 | 95.2 | 96.9 | 96.7 | 95.8 | 96.8 | 96.1 | 95.7 | 96.5 | 95.3 | 96.6 | 96.2 | 95.1 | 95.7 | 95.4 | 96.0 | 92.8 |
| 15 | Methylibium petroleiphilum PM1T | 96.4 | 96.1 | 93.8 | 95.0 | 95.7 | 95.7 | 95.5 | 94.7 | 94.6 | 94.4 | 94.4 | 94.6 | 96.9 | 97.9 | 100.0 | 95.9 | 95.2 | 97.2 | 96.6 | 95.9 | 97.4 | 96.0 | 96.9 | 96.7 | 95.0 | 97.1 | 96.5 | 95.7 | 96.0 | 95.9 | 96.4 | 92.6 |
| 16 | Caldimonas tepidiphila YIM 730274T | 97.3 | 97.2 | 94.8 | 95.7 | 96.3 | 96.3 | 96.1 | 95.3 | 94.6 | 94.9 | 95.0 | 95.2 | 95.9 | 95.7 | 95.9 | 100.0 | 96.8 | 97.2 | 96.7 | 97.0 | 95.4 | 96.6 | 97.0 | 97.2 | 96.1 | 97.1 | 97.4 | 95.5 | 95.7 | 96.5 | 96.7 | 93.0 |
| 17 | Caldimonas thermodepolymerans DSM 15344T | 95.7 | 96.2 | 94.8 | 95.0 | 95.1 | 96.1 | 95.9 | 94.1 | 93.9 | 94.2 | 94.3 | 94.4 | 95.8 | 95.2 | 95.2 | 96.8 | 100.0 | 97.1 | 96.8 | 97.5 | 94.7 | 95.4 | 96.1 | 97.1 | 95.9 | 96.7 | 95.8 | 95.8 | 94.9 | 96.0 | 96.6 | 92.4 |
| 18 | Caldimonas brevitalea DSM 7029T | 96.9 | 97.2 | 95.0 | 95.9 | 96.3 | 97.0 | 96.7 | 95.2 | 95.2 | 95.7 | 95.6 | 95.4 | 97.3 | 96.9 | 97.2 | 97.2 | 97.1 | 100.0 | 97.4 | 97.7 | 97.1 | 96.9 | 97.3 | 97.4 | 96.4 | 97.4 | 97.1 | 95.9 | 96.5 | 96.8 | 97.5 | 93.0 |
| 19 | Caldimonas caldifontis BCRC 80649T | 95.7 | 96.5 | 94.1 | 95.2 | 95.8 | 96.3 | 96.2 | 94.6 | 94.4 | 95.0 | 94.9 | 94.9 | 97.2 | 96.7 | 96.6 | 96.7 | 96.8 | 97.4 | 100.0 | 99.1 | 96.4 | 96.1 | 96.3 | 96.7 | 96.9 | 97.3 | 96.0 | 97.0 | 95.8 | 96.3 | 96.7 | 93.2 |
| 20 | Caldimonas manganoxidans ATCC BAA-369T | 96.2 | 97.0 | 95.4 | 96.3 | 96.7 | 96.7 | 96.7 | 96.1 | 96.0 | 96.3 | 96.2 | 96.3 | 96.3 | 95.8 | 95.9 | 97.0 | 97.5 | 97.7 | 99.1 | 100.0 | 95.6 | 96.7 | 96.4 | 97.5 | 96.8 | 97.3 | 96.8 | 97.2 | 97.1 | 97.0 | 97.1 | 94.4 |
| 21 | Piscinibacter gummiphilus NS21T | 96.3 | 95.6 | 94.3 | 95.7 | 95.7 | 95.9 | 95.8 | 94.3 | 94.3 | 94.7 | 94.6 | 94.3 | 96.9 | 96.8 | 97.4 | 95.4 | 94.7 | 97.1 | 96.4 | 95.6 | 100.0 | 96.3 | 96.9 | 96.5 | 94.8 | 96.4 | 96.0 | 95.3 | 95.9 | 95.6 | 96.0 | 91.6 |
| 22 | Piscinibacter sakaiensis 201-F6T | 96.2 | 98.1 | 95.5 | 96.4 | 96.5 | 96.7 | 96.5 | 95.4 | 95.3 | 95.7 | 95.7 | 95.3 | 95.6 | 96.1 | 96.0 | 96.6 | 95.4 | 96.9 | 96.1 | 96.7 | 96.4 | 100.0 | 97.4 | 97.4 | 96.1 | 97.4 | 96.8 | 96.3 | 96.7 | 97.5 | 98.0 | 92.2 |
| 23 | Piscinibacter defluvii SH-1T | 96.5 | 96.9 | 94.6 | 96.0 | 95.7 | 96.3 | 96.1 | 94.8 | 94.8 | 94.6 | 94.6 | 94.8 | 96.0 | 95.7 | 96.9 | 97.0 | 96.1 | 97.3 | 96.3 | 96.4 | 96.9 | 97.4 | 100.0 | 98.1 | 95.3 | 96.9 | 96.9 | 95.5 | 96.2 | 96.9 | 97.1 | 91.8 |
| 24 | Azohydromonas sediminis SYSU G00088T | 97.0 | 97.1 | 95.4 | 96.1 | 96.2 | 96.7 | 96.6 | 95.2 | 95.2 | 95.0 | 95.0 | 95.3 | 96.5 | 96.5 | 96.7 | 97.2 | 97.1 | 97.4 | 96.7 | 97.5 | 96.5 | 97.4 | 98.1 | 100.0 | 95.9 | 97.5 | 97.4 | 95.7 | 96.7 | 96.8 | 97.2 | 92.0 |
| 25 | Azohydromonas australica DSM 1124T | 95.0 | 96.3 | 94.7 | 96.1 | 96.1 | 96.1 | 96.1 | 95.6 | 95.6 | 95.7 | 95.6 | 95.7 | 95.5 | 95.3 | 95.0 | 96.1 | 95.9 | 96.4 | 96.9 | 96.8 | 94.8 | 96.1 | 95.3 | 95.9 | 100.0 | 97.5 | 95.7 | 96.3 | 95.8 | 96.3 | 96.1 | 93.9 |
| 26 | Azohydromonas lata NBRC 102462T | 96.9 | 97.7 | 96.1 | 97.2 | 97.6 | 97.5 | 97.5 | 97.0 | 97.1 | 96.5 | 96.6 | 97.2 | 96.6 | 96.6 | 97.1 | 97.1 | 96.7 | 97.4 | 97.3 | 97.3 | 96.4 | 97.4 | 96.9 | 97.5 | 97.5 | 100.0 | 96.9 | 97.4 | 97.7 | 97.9 | 98.0 | 94.6 |
| 27 | Rubrivivax pictus W35T | 98.0 | 97.9 | 95.5 | 96.1 | 97.0 | 97.0 | 96.7 | 95.7 | 95.4 | 95.7 | 95.6 | 95.7 | 96.7 | 96.2 | 96.5 | 97.4 | 95.8 | 97.1 | 96.0 | 96.8 | 96.0 | 96.8 | 96.9 | 97.4 | 95.7 | 96.9 | 100.0 | 96.0 | 97.3 | 96.8 | 97.6 | 93.2 |
| 28 | Rubrivivax albus ICH-3T | 95.4 | 97.1 | 94.8 | 95.4 | 95.9 | 96.3 | 96.0 | 94.4 | 94.6 | 94.8 | 95.0 | 95.0 | 96.3 | 95.1 | 95.7 | 95.5 | 95.8 | 95.9 | 97.0 | 97.2 | 95.3 | 96.3 | 95.5 | 95.7 | 96.3 | 97.4 | 96.0 | 100.0 | 95.0 | 97.6 | 97.9 | 92.4 |
| 29 | Rubrivivax rivuli KYPY4T | 97.0 | 96.7 | 92.9 | 95.2 | 95.2 | 95.0 | 94.8 | 93.5 | 93.9 | 93.9 | 93.8 | 93.2 | 94.9 | 95.7 | 96.0 | 95.7 | 94.9 | 96.5 | 95.8 | 97.1 | 95.9 | 96.7 | 96.2 | 96.7 | 95.8 | 97.7 | 97.3 | 95.0 | 100.0 | 95.2 | 94.7 | 90.8 |
| 30 | Rubrivivax gelatinosus DSM 1709T | 96.1 | 97.8 | 95.3 | 96.4 | 97.1 | 97.5 | 97.4 | 95.9 | 95.8 | 95.9 | 96.1 | 96.5 | 96.3 | 95.4 | 95.9 | 96.5 | 96.0 | 96.8 | 96.3 | 97.0 | 95.6 | 97.5 | 96.9 | 96.8 | 96.3 | 97.9 | 96.8 | 97.6 | 95.2 | 100.0 | 99.9 | 92.2 |
| 31 | Rubrivivax benzoatilyticus JA2T | 96.9 | 98.6 | 97.0 | 97.8 | 98.0 | 98.6 | 98.5 | 97.2 | 97.2 | 97.3 | 97.6 | 97.9 | 97.3 | 96.0 | 96.4 | 96.7 | 96.6 | 97.5 | 96.7 | 97.1 | 96.0 | 98.0 | 97.1 | 97.2 | 96.1 | 98.0 | 97.6 | 97.9 | 94.7 | 99.9 | 100.0 | 93.6 |
| 32 | Comamonas terrigena NBRC 13299T | 92.5 | 93.0 | 91.4 | 92.6 | 92.4 | 92.4 | 92.5 | 92.7 | 92.6 | 92.4 | 92.2 | 92.2 | 92.2 | 92.8 | 92.6 | 93.0 | 92.4 | 93.0 | 93.2 | 94.4 | 91.6 | 92.2 | 91.8 | 92.0 | 94.0 | 94.6 | 93.2 | 92.4 | 90.8 | 92.2 | 93.6 | 100.0 |

## Slide 3
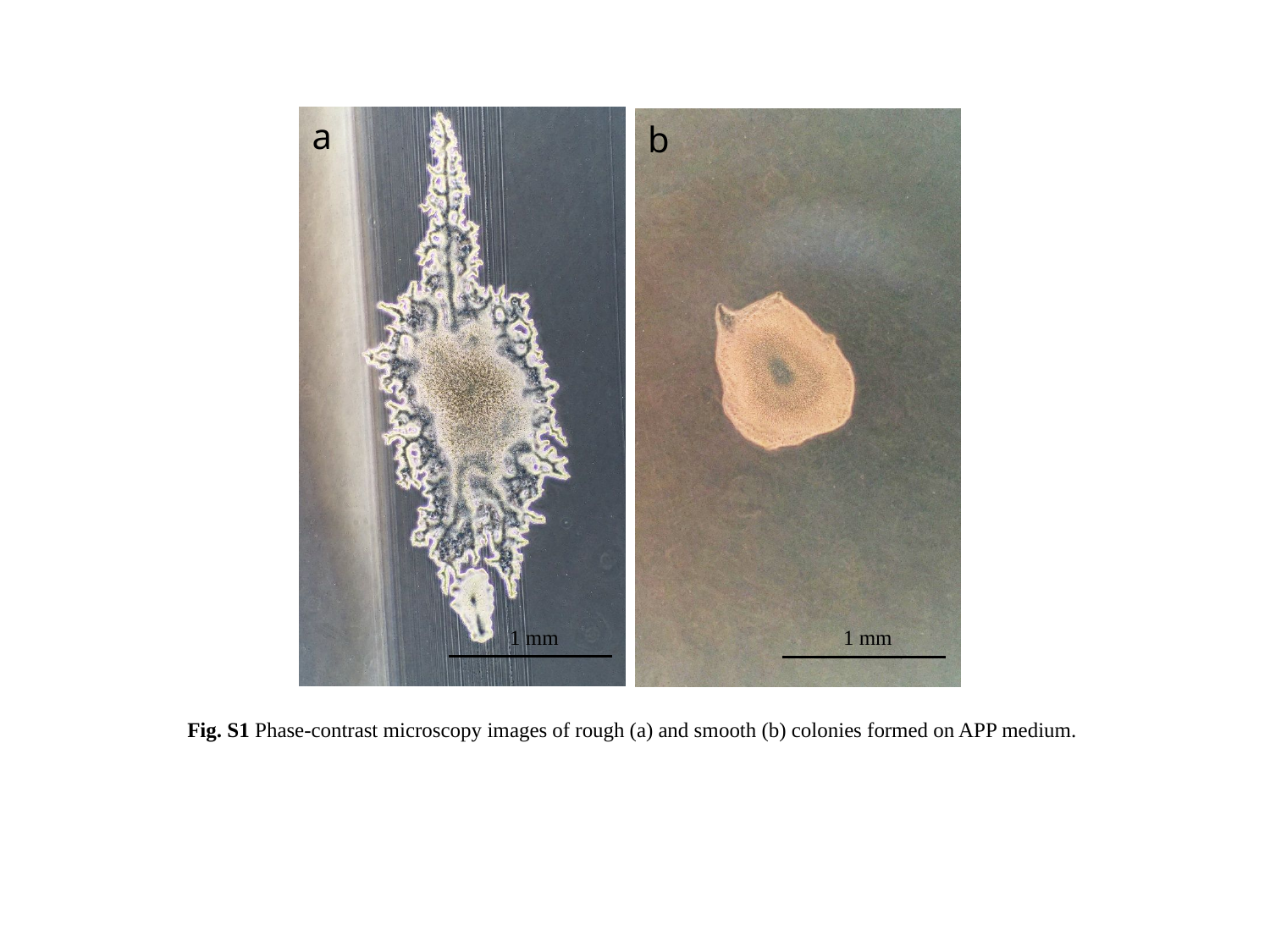

a
1 mm
1 mm
b
Fig. S1 Phase-contrast microscopy images of rough (a) and smooth (b) colonies formed on APP medium.

## Slide 4
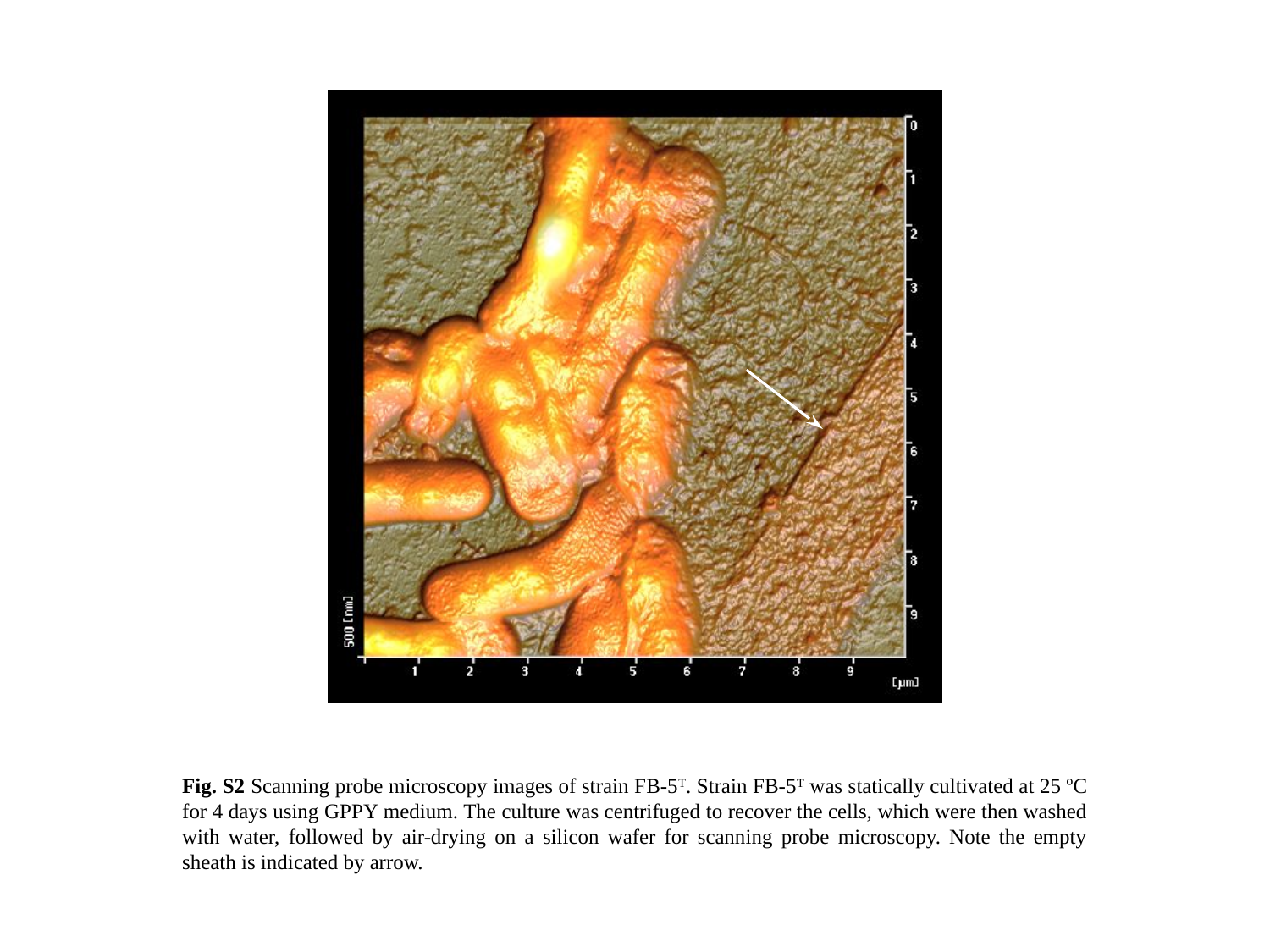

Fig. S2 Scanning probe microscopy images of strain FB-5T. Strain FB-5T was statically cultivated at 25 ºC for 4 days using GPPY medium. The culture was centrifuged to recover the cells, which were then washed with water, followed by air-drying on a silicon wafer for scanning probe microscopy. Note the empty sheath is indicated by arrow.

## Slide 5
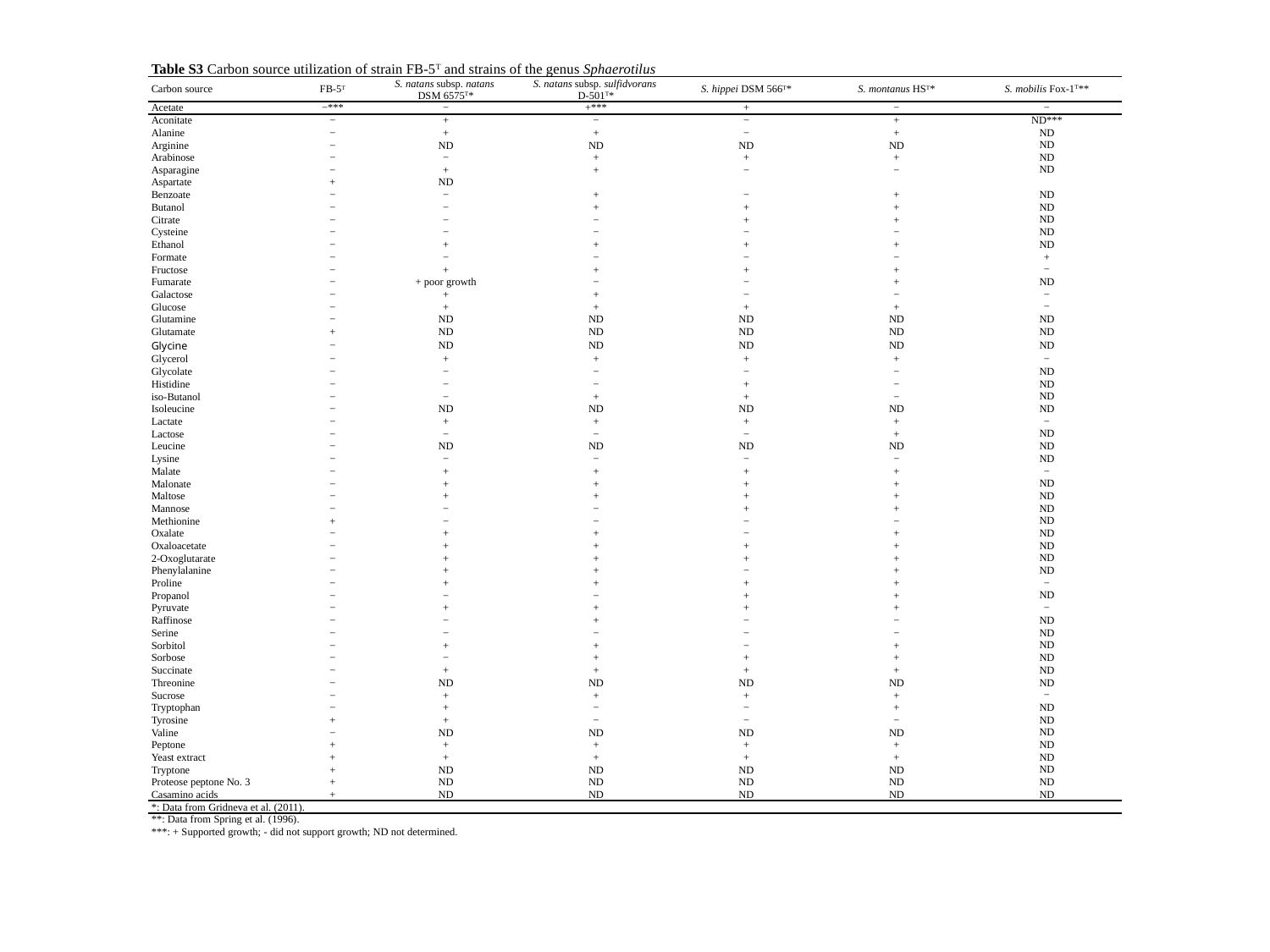

| Table S3 Carbon source utilization of strain FB-5T and strains of the genus Sphaerotilus | | | | | | |
| --- | --- | --- | --- | --- | --- | --- |
| Carbon source | FB-5T | S. natans subsp. natans DSM 6575T\* | S. natans subsp. sulfidvorans D-501T\* | S. hippei DSM 566T\* | S. montanus HST\* | S. mobilis Fox-1T\*\* |
| Acetate | −\*\*\* | − | +\*\*\* | + | − | − |
| Aconitate | − | + | − | − | + | nd\*\*\* |
| Alanine | − | + | + | − | + | nd |
| Arginine | − | ND | ND | ND | ND | ND |
| Arabinose | − | − | + | + | + | nd |
| Asparagine | − | + | + | − | − | nd |
| Aspartate | + | ND | | | | |
| Benzoate | − | − | + | − | + | nd |
| Butanol | − | − | + | + | + | nd |
| Citrate | − | − | − | + | + | nd |
| Cysteine | − | − | − | − | − | nd |
| Ethanol | − | + | + | + | + | nd |
| Formate | − | − | − | − | − | + |
| Fructose | − | + | + | + | + | − |
| Fumarate | − | + poor growth | − | − | + | nd |
| Galactose | − | + | + | − | − | − |
| Glucose | − | + | + | + | + | − |
| Glutamine | − | ND | ND | ND | ND | ND |
| Glutamate | + | ND | ND | ND | ND | ND |
| Glycine | − | ND | ND | ND | ND | ND |
| Glycerol | − | + | + | + | + | − |
| Glycolate | − | − | − | − | − | nd |
| Histidine | − | − | − | + | − | nd |
| iso-Butanol | − | − | + | + | − | nd |
| Isoleucine | − | ND | ND | ND | ND | ND |
| Lactate | − | + | + | + | + | − |
| Lactose | − | − | − | − | + | nd |
| Leucine | − | ND | ND | ND | ND | ND |
| Lysine | − | − | − | − | − | nd |
| Malate | − | + | + | + | + | − |
| Malonate | − | + | + | + | + | nd |
| Maltose | − | + | + | + | + | nd |
| Mannose | − | − | − | + | + | Nd |
| Methionine | + | − | − | − | − | nd |
| Oxalate | − | + | + | − | + | nd |
| Oxaloacetate | − | + | + | + | + | nd |
| 2-Oxoglutarate | − | + | + | + | + | nd |
| Phenylalanine | − | + | + | − | + | nd |
| Proline | − | + | + | + | + | − |
| Propanol | − | − | − | + | + | nd |
| Pyruvate | − | + | + | + | + | − |
| Raffinose | − | − | + | − | − | nd |
| Serine | − | − | − | − | − | nd |
| Sorbitol | − | + | + | − | + | nd |
| Sorbose | − | − | + | + | + | nd |
| Succinate | − | + | + | + | + | nd |
| Threonine | − | ND | ND | ND | ND | ND |
| Sucrose | − | + | + | + | + | − |
| Tryptophan | − | + | − | − | + | ND |
| Tyrosine | + | + | − | − | − | ND |
| Valine | − | ND | ND | ND | ND | ND |
| Peptone | + | + | + | + | + | ND |
| Yeast extract | + | + | + | + | + | ND |
| Tryptone | + | ND | ND | ND | ND | ND |
| Proteose peptone No. 3 | + | ND | ND | ND | ND | ND |
| Casamino acids | + | ND | ND | ND | ND | ND |
| \*: Data from Gridneva et al. (2011). | | | | | | |
| \*\*: Data from Spring et al. (1996). | | | | | | |
| \*\*\*: + Supported growth; - did not support growth; ND not determined. | | | | | | |

## Slide 6
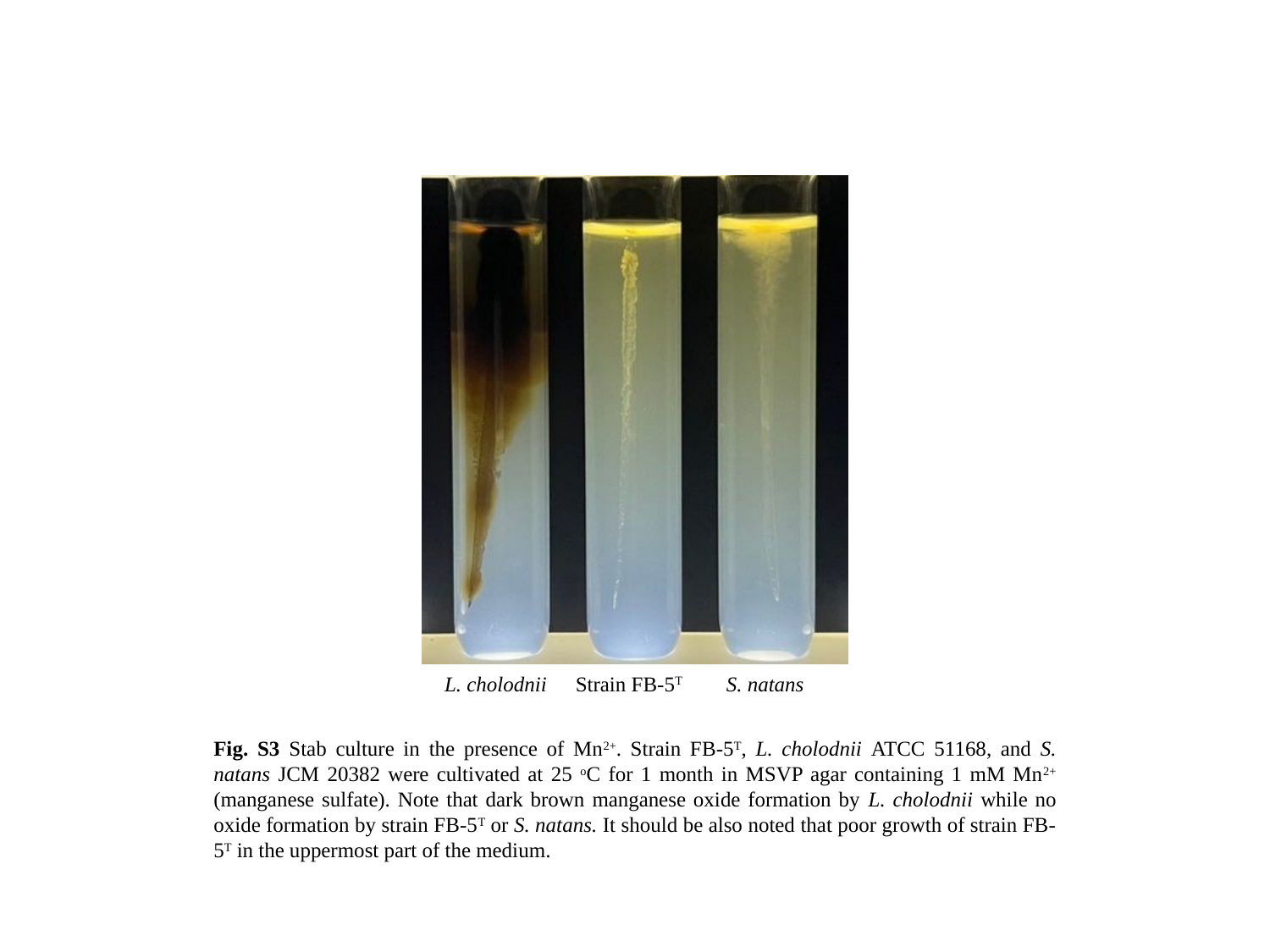

L. cholodnii
Strain FB-5T
S. natans
Fig. S3 Stab culture in the presence of Mn2+. Strain FB-5T, L. cholodnii ATCC 51168, and S. natans JCM 20382 were cultivated at 25 oC for 1 month in MSVP agar containing 1 mM Mn2+ (manganese sulfate). Note that dark brown manganese oxide formation by L. cholodnii while no oxide formation by strain FB-5T or S. natans. It should be also noted that poor growth of strain FB-5T in the uppermost part of the medium.

## Slide 7
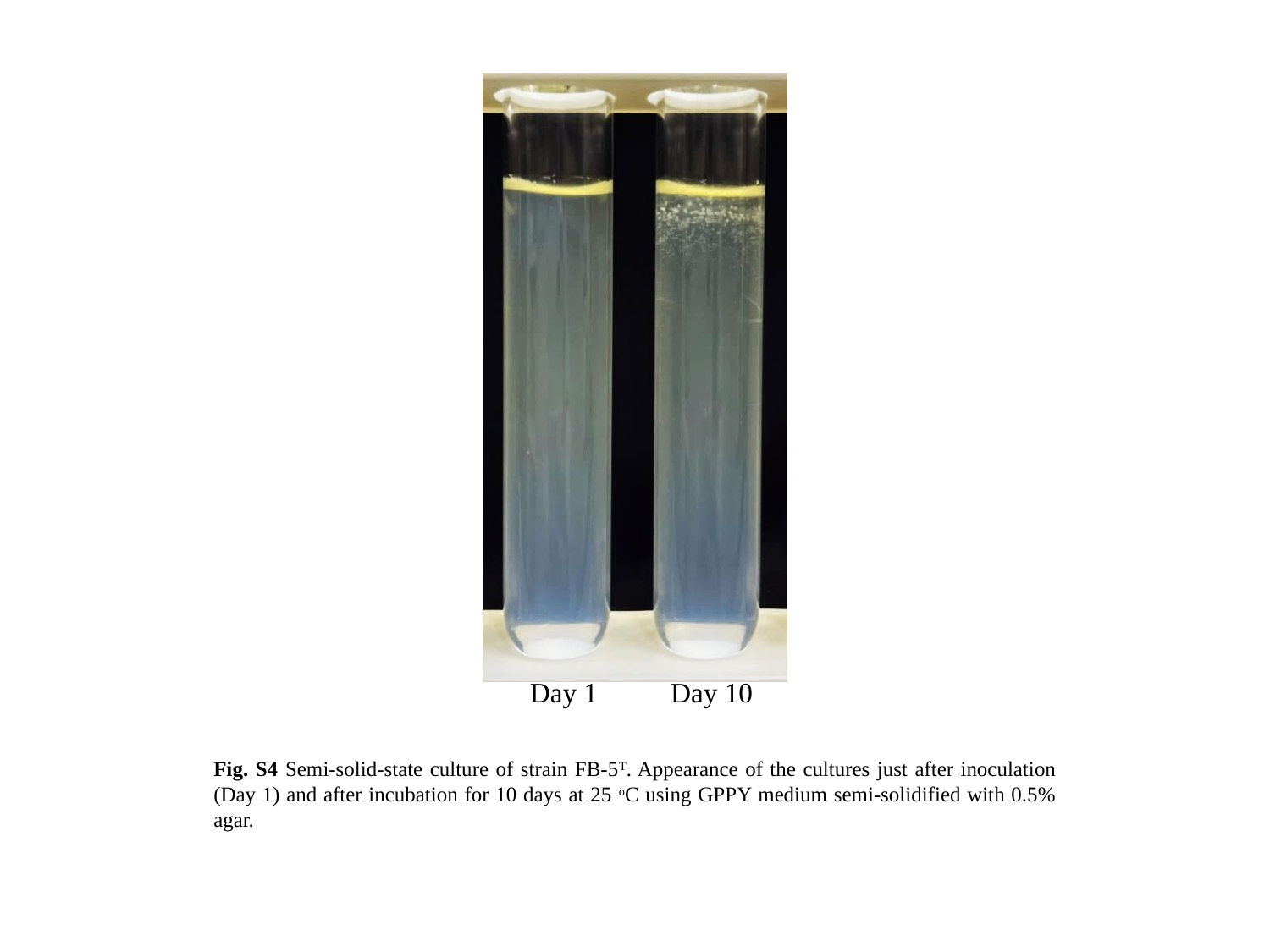

Day 1
Day 10
Fig. S4 Semi-solid-state culture of strain FB-5T. Appearance of the cultures just after inoculation (Day 1) and after incubation for 10 days at 25 oC using GPPY medium semi-solidified with 0.5% agar.

## Slide 8
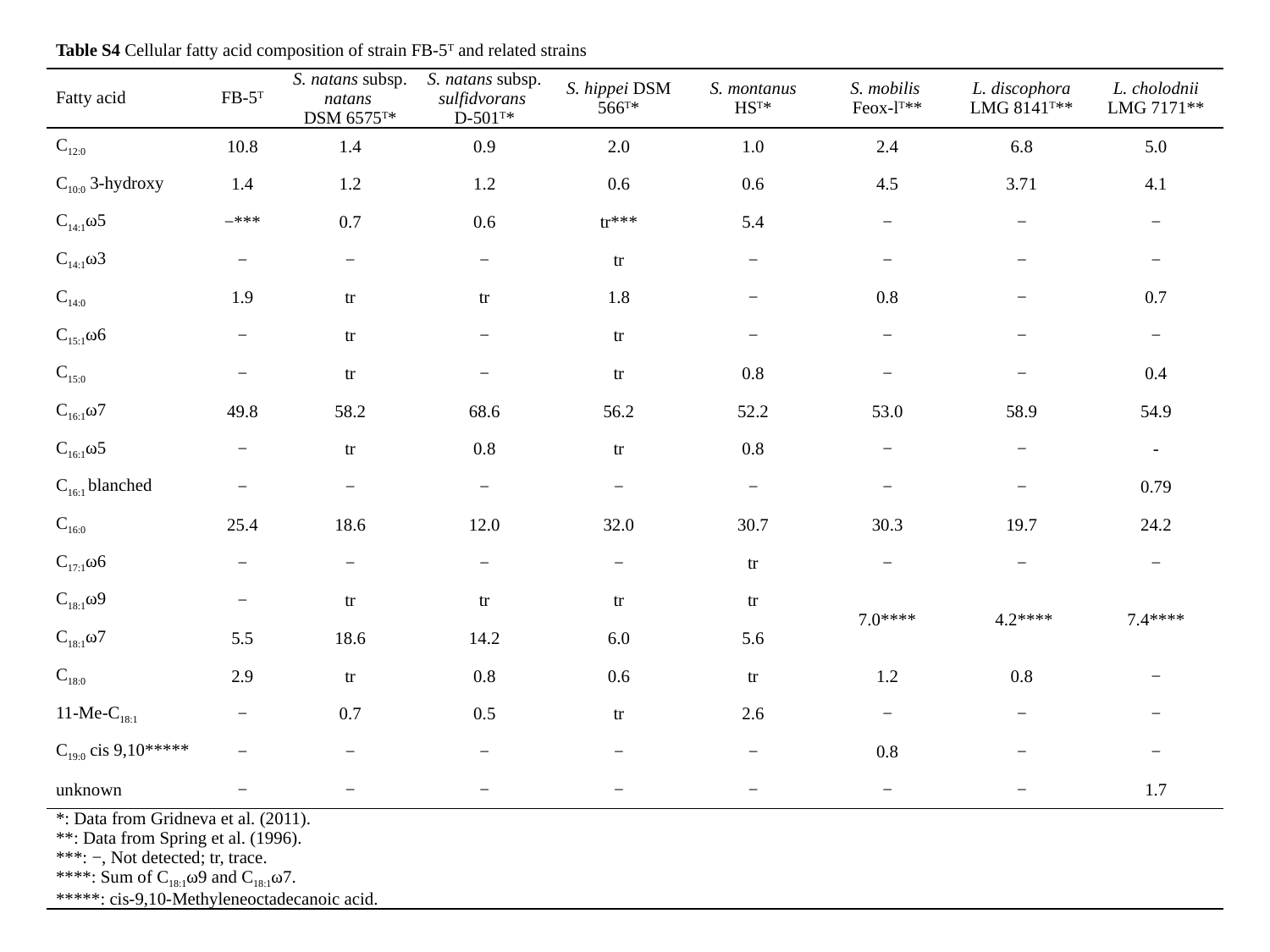

| Table S4 Cellular fatty acid composition of strain FB-5T and related strains | | | | | | | | |
| --- | --- | --- | --- | --- | --- | --- | --- | --- |
| Fatty acid | FB-5T | S. natans subsp. natans DSM 6575T\* | S. natans subsp. sulfidvorans D-501T\* | S. hippei DSM 566T\* | S. montanus HST\* | S. mobilis Feox-lT\*\* | L. discophora LMG 8141T\*\* | L. cholodnii LMG 7171\*\* |
| C12:0 | 10.8 | 1.4 | 0.9 | 2.0 | 1.0 | 2.4 | 6.8 | 5.0 |
| C10:0 3-hydroxy | 1.4 | 1.2 | 1.2 | 0.6 | 0.6 | 4.5 | 3.71 | 4.1 |
| C14:1ω5 | −\*\*\* | 0.7 | 0.6 | tr\*\*\* | 5.4 | − | − | − |
| C14:1ω3 | − | − | − | tr | − | − | − | − |
| C14:0 | 1.9 | tr | tr | 1.8 | − | 0.8 | − | 0.7 |
| C15:1ω6 | − | tr | − | tr | − | − | − | − |
| C15:0 | − | tr | − | tr | 0.8 | − | − | 0.4 |
| C16:1ω7 | 49.8 | 58.2 | 68.6 | 56.2 | 52.2 | 53.0 | 58.9 | 54.9 |
| C16:1ω5 | − | tr | 0.8 | tr | 0.8 | − | − | - |
| C16:1 blanched | − | − | − | − | − | − | − | 0.79 |
| C16:0 | 25.4 | 18.6 | 12.0 | 32.0 | 30.7 | 30.3 | 19.7 | 24.2 |
| C17:1ω6 | − | − | − | − | tr | − | − | − |
| C18:1ω9 | − | tr | tr | tr | tr | 7.0\*\*\*\* | 4.2\*\*\*\* | 7.4\*\*\*\* |
| C18:1ω7 | 5.5 | 18.6 | 14.2 | 6.0 | 5.6 | | | |
| C18:0 | 2.9 | tr | 0.8 | 0.6 | tr | 1.2 | 0.8 | − |
| 11-Me-C18:1 | − | 0.7 | 0.5 | tr | 2.6 | − | − | − |
| C19:0 cis 9,10\*\*\*\*\* | − | − | − | − | − | 0.8 | − | − |
| unknown | − | − | − | − | − | − | − | 1.7 |
| \*: Data from Gridneva et al. (2011). \*\*: Data from Spring et al. (1996). \*\*\*: −, Not detected; tr, trace. \*\*\*\*: Sum of C18:1ω9 and C18:1ω7. \*\*\*\*\*: cis-9,10-Methyleneoctadecanoic acid. | | | | | | | | |

## Slide 9
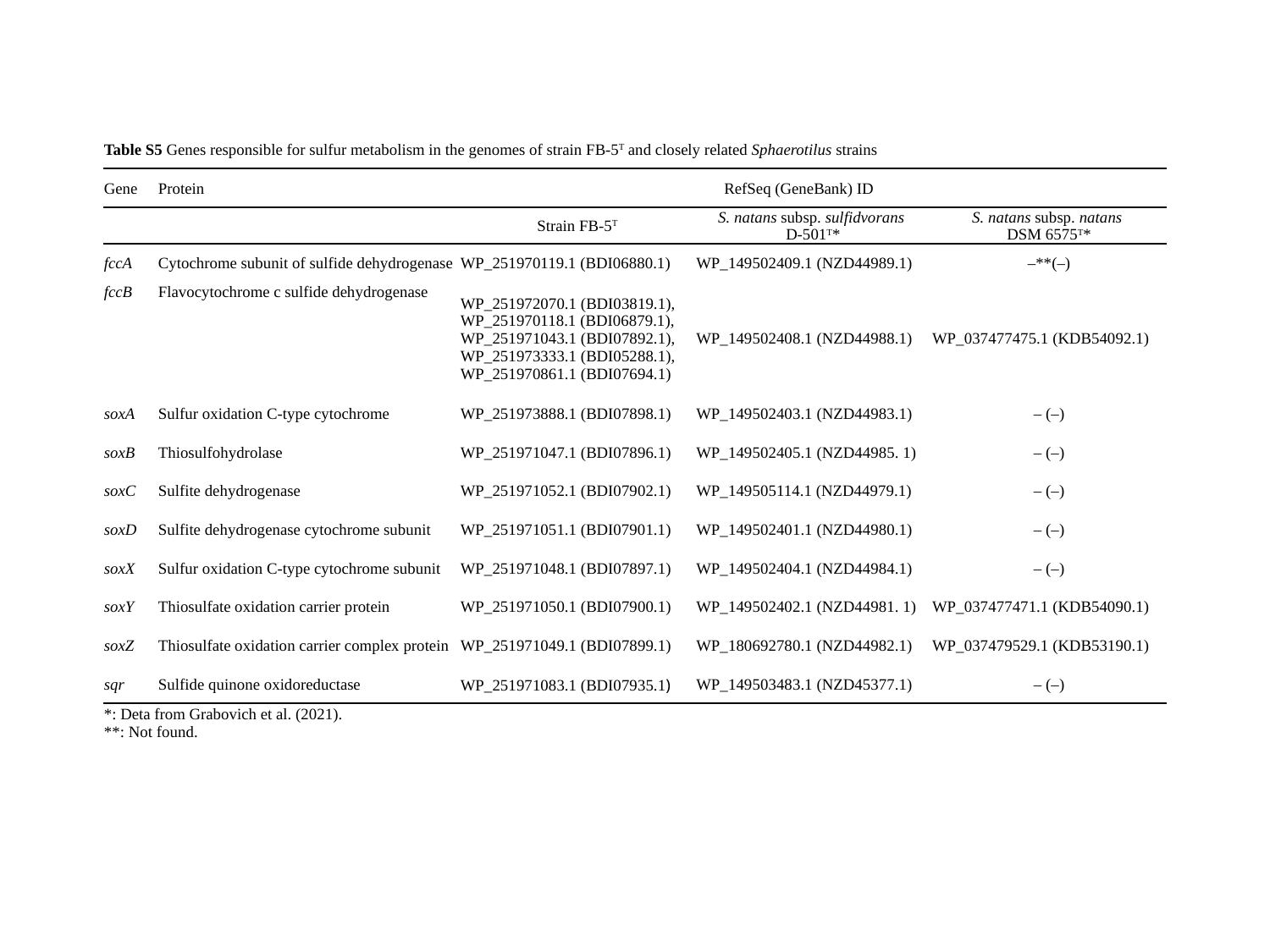

| Table S5 Genes responsible for sulfur metabolism in the genomes of strain FB-5T and closely related Sphaerotilus strains | | | | | |
| --- | --- | --- | --- | --- | --- |
| Gene | Protein | RefSeq (GeneBank) ID | | | RefSeq (GeneBank) ID |
| | | | Strain FB-5T | S. natans subsp. sulfidvorans D-501T\* | S. natans subsp. natans DSM 6575T\* |
| fccA | Cytochrome subunit of sulfide dehydrogenase | | WP\_251970119.1 (BDI06880.1) | WP\_149502409.1 (NZD44989.1) | ‒\*\*(‒) |
| fccB | Flavocytochrome c sulfide dehydrogenase | | WP\_251972070.1 (BDI03819.1), WP\_251970118.1 (BDI06879.1), WP\_251971043.1 (BDI07892.1), WP\_251973333.1 (BDI05288.1), WP\_251970861.1 (BDI07694.1) | WP\_149502408.1 (NZD44988.1) | WP\_037477475.1 (KDB54092.1) |
| soxA | Sulfur oxidation C-type cytochrome | | WP\_251973888.1 (BDI07898.1) | WP\_149502403.1 (NZD44983.1) | ‒ (‒) |
| soxB | Thiosulfohydrolase | | WP\_251971047.1 (BDI07896.1) | WP\_149502405.1 (NZD44985. 1) | ‒ (‒) |
| soxC | Sulfite dehydrogenase | | WP\_251971052.1 (BDI07902.1) | WP\_149505114.1 (NZD44979.1) | ‒ (‒) |
| soxD | Sulfite dehydrogenase cytochrome subunit | | WP\_251971051.1 (BDI07901.1) | WP\_149502401.1 (NZD44980.1) | ‒ (‒) |
| soxX | Sulfur oxidation C-type cytochrome subunit | | WP\_251971048.1 (BDI07897.1) | WP\_149502404.1 (NZD44984.1) | ‒ (‒) |
| soxY | Thiosulfate oxidation carrier protein | | WP\_251971050.1 (BDI07900.1) | WP\_149502402.1 (NZD44981. 1) | WP\_037477471.1 (KDB54090.1) |
| soxZ | Thiosulfate oxidation carrier complex protein | | WP\_251971049.1 (BDI07899.1) | WP\_180692780.1 (NZD44982.1) | WP\_037479529.1 (KDB53190.1) |
| sqr | Sulfide quinone oxidoreductase | | WP\_251971083.1 (BDI07935.1) | WP\_149503483.1 (NZD45377.1) | ‒ (‒) |
| \*: Deta from Grabovich et al. (2021). \*\*: Not found. | | | | | |

## Slide 10
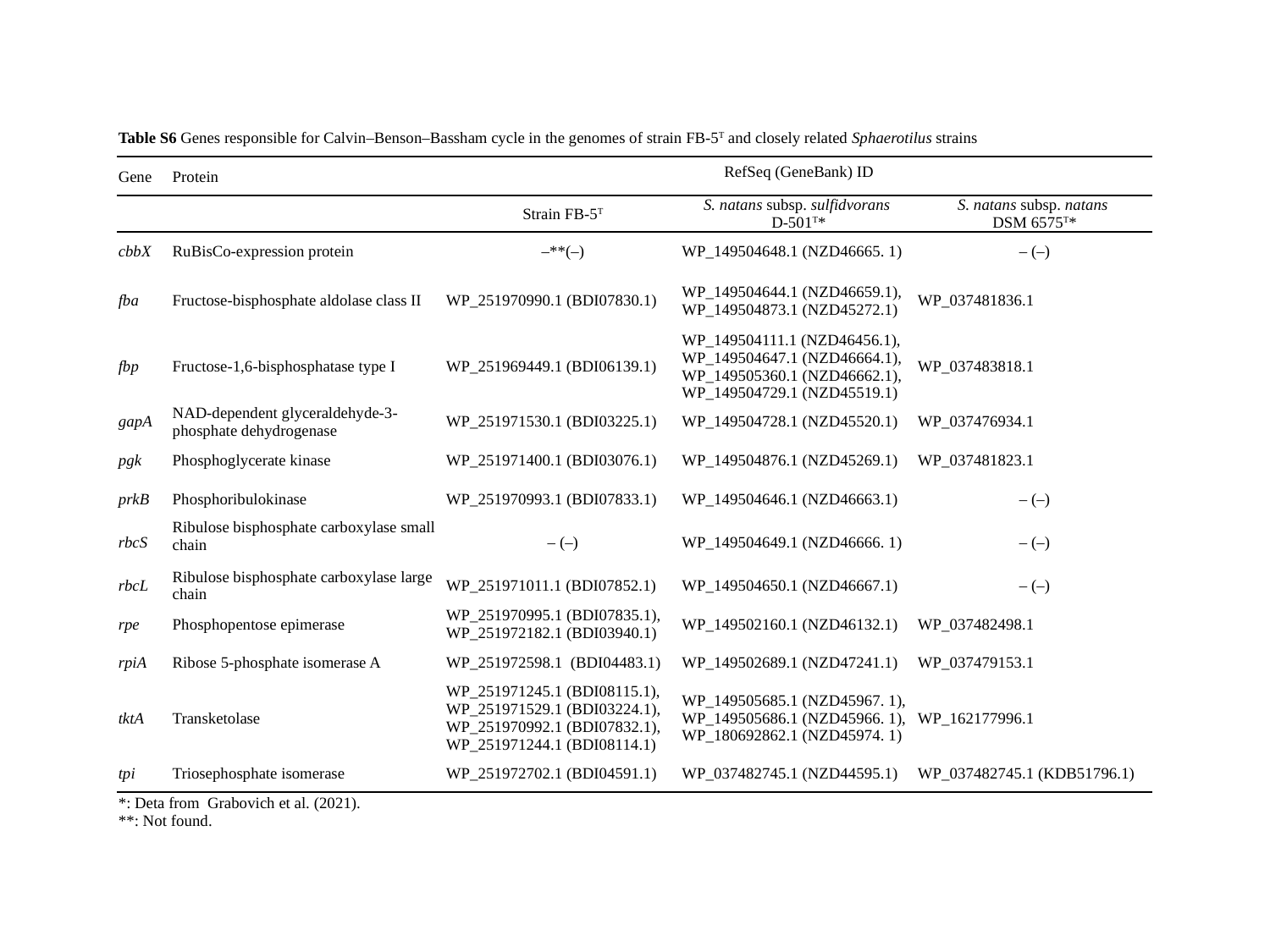

| Table S6 Genes responsible for Calvin–Benson–Bassham cycle in the genomes of strain FB-5T and closely related Sphaerotilus strains | | | | |
| --- | --- | --- | --- | --- |
| Gene | Protein | RefSeq (GeneBank) ID | | RefSeq (GeneBank) ID |
| | | Strain FB-5T | S. natans subsp. sulfidvorans D-501T\* | S. natans subsp. natans DSM 6575T\* |
| cbbX | RuBisCo-expression protein | ‒\*\*(‒) | WP\_149504648.1 (NZD46665. 1) | ‒ (‒) |
| fba | Fructose-bisphosphate aldolase class II | WP\_251970990.1 (BDI07830.1) | WP\_149504644.1 (NZD46659.1), WP\_149504873.1 (NZD45272.1) | WP\_037481836.1 |
| fbp | Fructose-1,6-bisphosphatase type I | WP\_251969449.1 (BDI06139.1) | WP\_149504111.1 (NZD46456.1), WP\_149504647.1 (NZD46664.1), WP\_149505360.1 (NZD46662.1), WP\_149504729.1 (NZD45519.1) | WP\_037483818.1 |
| gapA | NAD-dependent glyceraldehyde-3-phosphate dehydrogenase | WP\_251971530.1 (BDI03225.1) | WP\_149504728.1 (NZD45520.1) | WP\_037476934.1 |
| pgk | Phosphoglycerate kinase | WP\_251971400.1 (BDI03076.1) | WP\_149504876.1 (NZD45269.1) | WP\_037481823.1 |
| prkB | Phosphoribulokinase | WP\_251970993.1 (BDI07833.1) | WP\_149504646.1 (NZD46663.1) | ‒ (‒) |
| rbcS | Ribulose bisphosphate carboxylase small chain | ‒ (‒) | WP\_149504649.1 (NZD46666. 1) | ‒ (‒) |
| rbcL | Ribulose bisphosphate carboxylase large chain | WP\_251971011.1 (BDI07852.1) | WP\_149504650.1 (NZD46667.1) | ‒ (‒) |
| rpe | Phosphopentose epimerase | WP\_251970995.1 (BDI07835.1), WP\_251972182.1 (BDI03940.1) | WP\_149502160.1 (NZD46132.1) | WP\_037482498.1 |
| rpiA | Ribose 5-phosphate isomerase A | WP\_251972598.1 (BDI04483.1) | WP\_149502689.1 (NZD47241.1) | WP\_037479153.1 |
| tktA | Transketolase | WP\_251971245.1 (BDI08115.1), WP\_251971529.1 (BDI03224.1), WP\_251970992.1 (BDI07832.1), WP\_251971244.1 (BDI08114.1) | WP\_149505685.1 (NZD45967. 1), WP\_149505686.1 (NZD45966. 1), WP\_180692862.1 (NZD45974. 1) | WP\_162177996.1 |
| tpi | Triosephosphate isomerase | WP\_251972702.1 (BDI04591.1) | WP\_037482745.1 (NZD44595.1) | WP\_037482745.1 (KDB51796.1) |
| \*: Deta from Grabovich et al. (2021). \*\*: Not found. | | | | |

## Slide 11
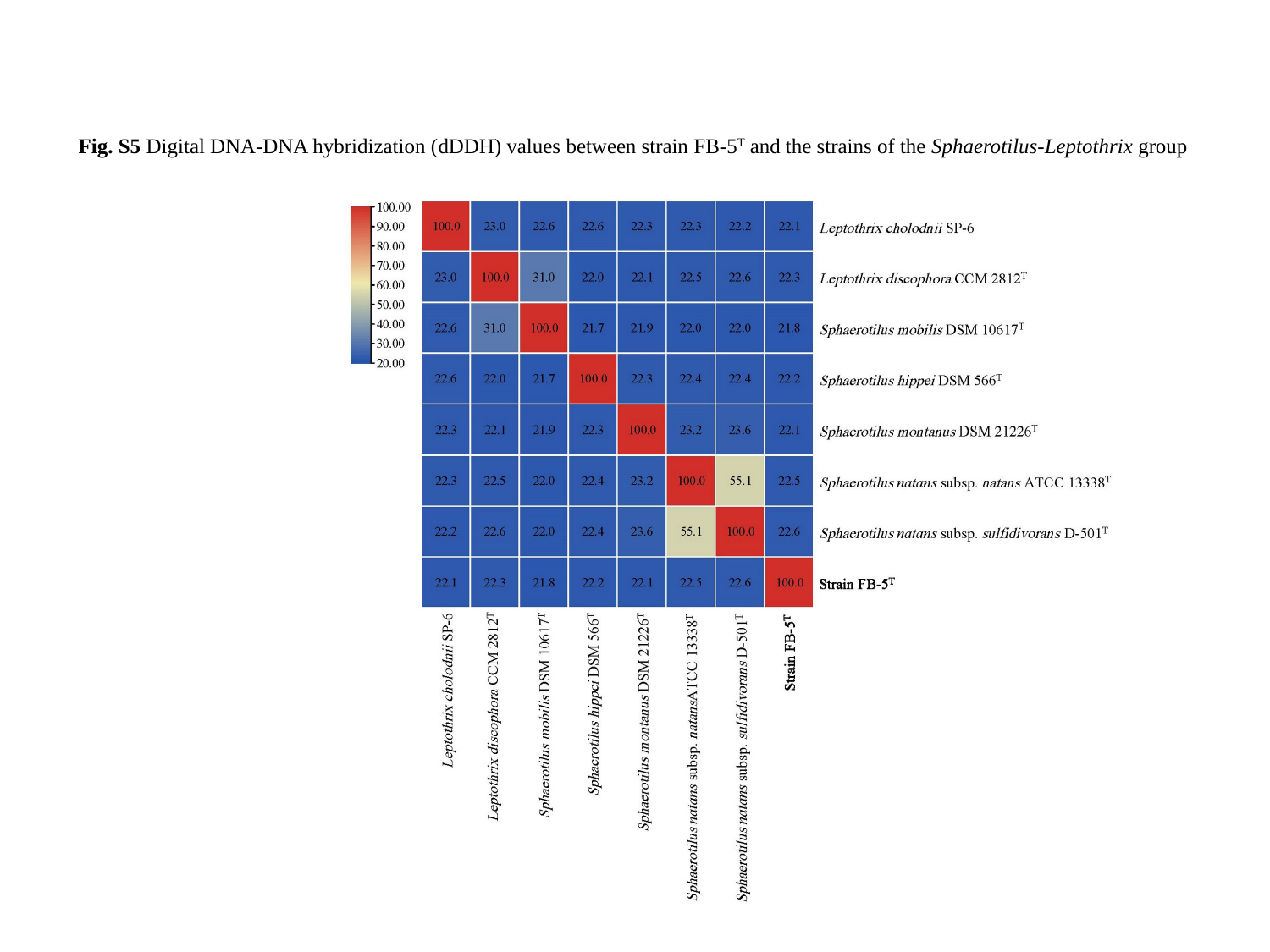

| Fig. S5 Digital DNA-DNA hybridization (dDDH) values between strain FB-5T and the strains of the Sphaerotilus-Leptothrix group |
| --- |

## Slide 12
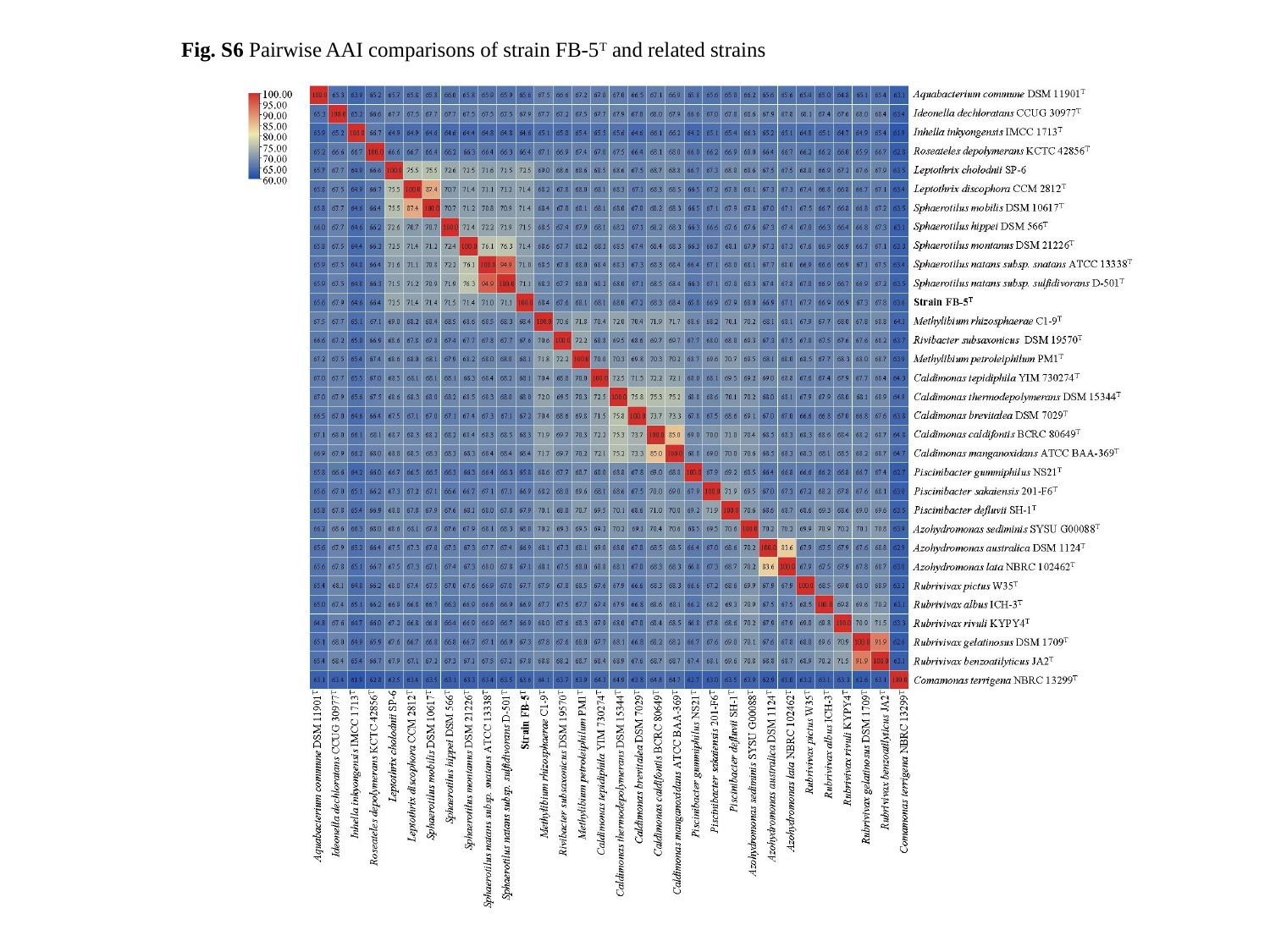

| Fig. S6 Pairwise AAI comparisons of strain FB-5T and related strains |
| --- |

## Slide 13
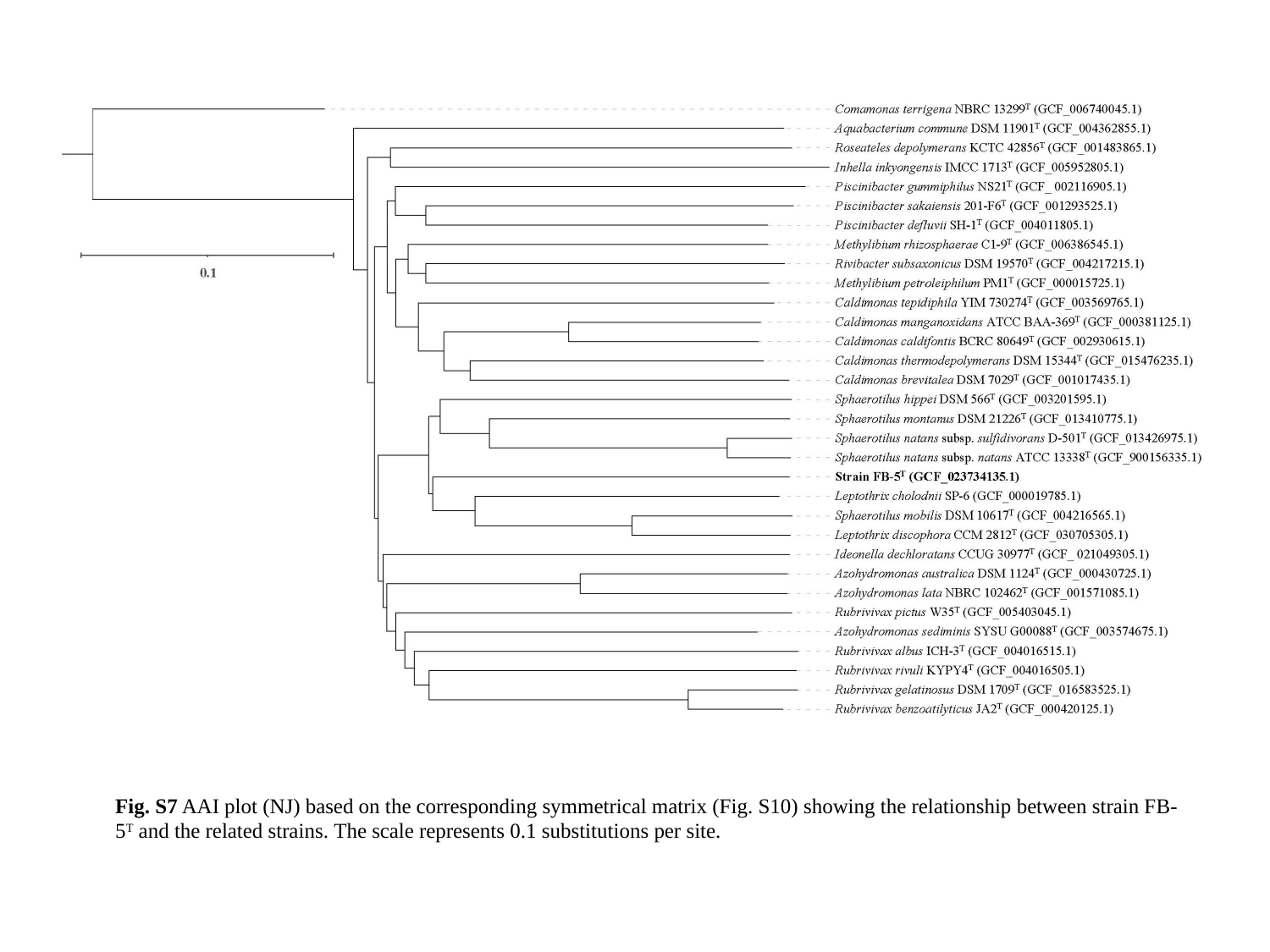

Fig. S7 AAI plot (NJ) based on the corresponding symmetrical matrix (Fig. S10) showing the relationship between strain FB-5T and the related strains. The scale represents 0.1 substitutions per site.

## Slide 14
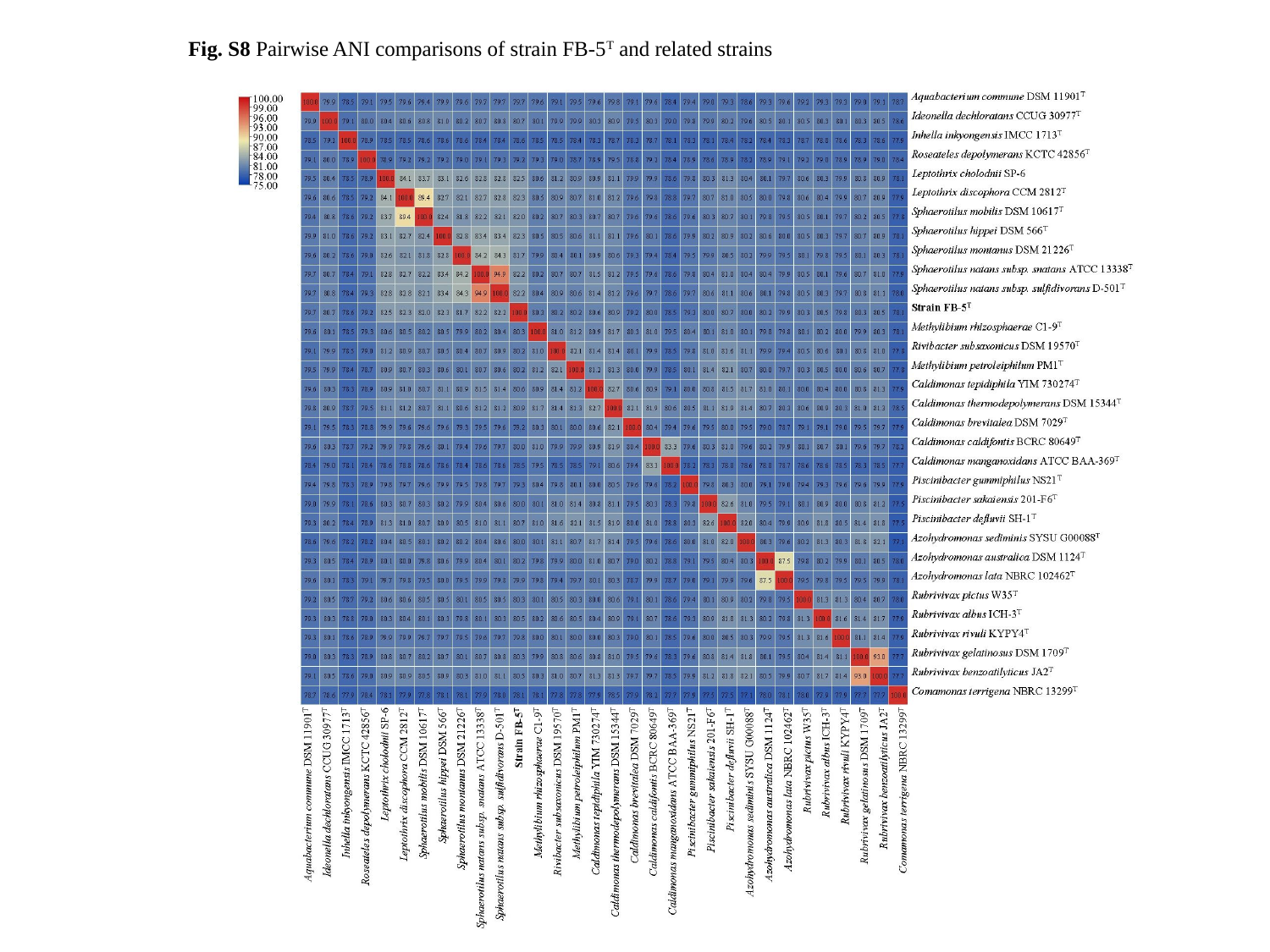

| Fig. S8 Pairwise ANI comparisons of strain FB-5T and related strains |
| --- |

## Slide 15
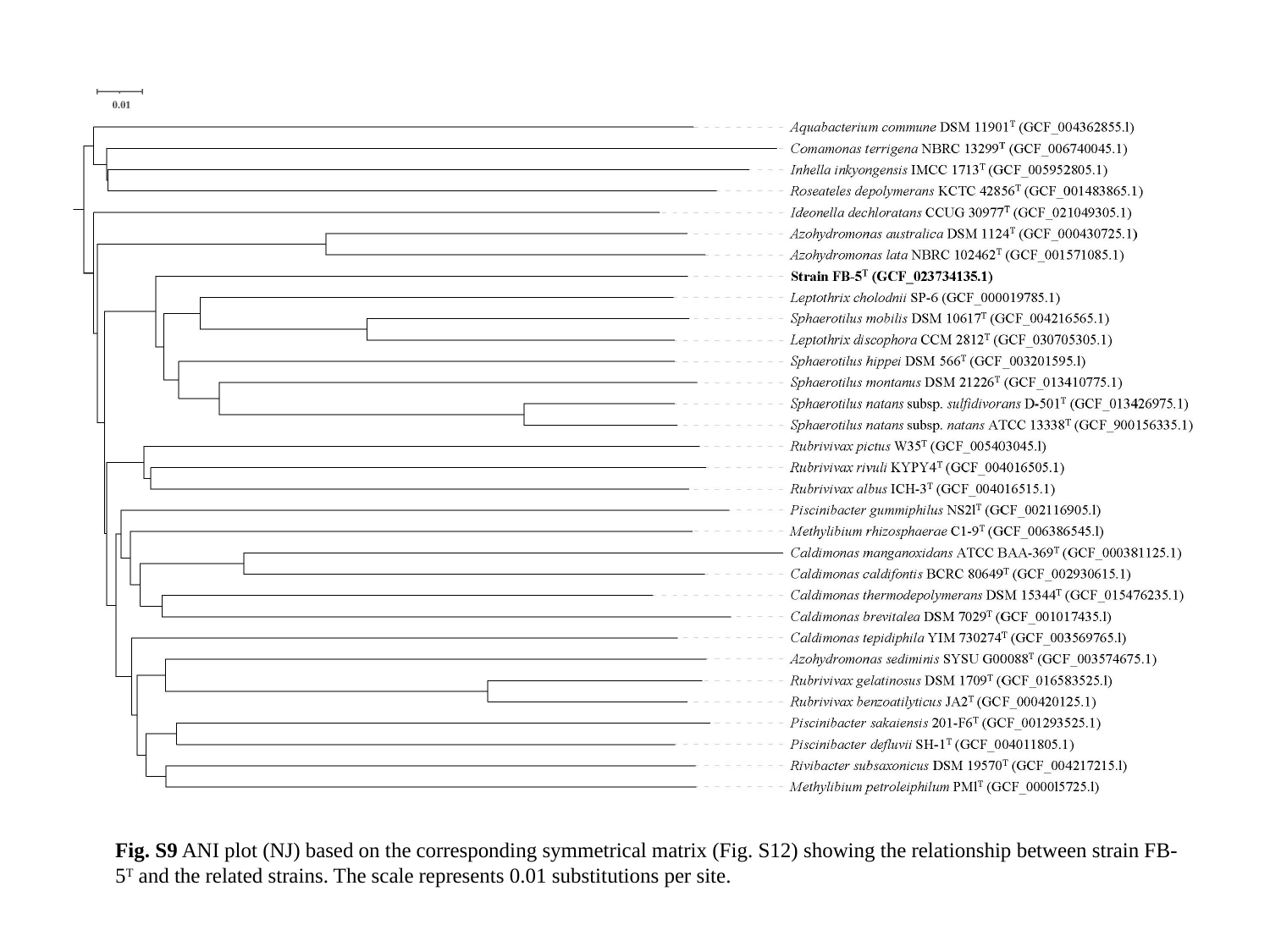

Fig. S9 ANI plot (NJ) based on the corresponding symmetrical matrix (Fig. S12) showing the relationship between strain FB-5T and the related strains. The scale represents 0.01 substitutions per site.
